# Natural *panax notoginseng*-derived nanovesicles trigger multiple cell death mechanisms and reprogram chemokine signaling to impede oral squamous cell carcinoma progression

**DOI:** 10.1186/s12951-026-04193-9

**Published:** 2026-03-06

**Authors:** Xiaohang Chen, Meifang Lin, Xuzheng Zhan, Genggeng Zheng, Shuoqi Lin, Liyu Huang, Chaochao Zhang, Yuxiang Yan, Hengyi Li, Zhaoyu Zhang, Xing Wang, Youguang Lu, Dali Zheng

**Affiliations:** 1 Fujian Key Laboratory of Oral Diseases, School and Hospital of Stomatology, Fujian Medical University, Fuzhou, China; 2 Department of Preventive Dentistry, School and Hospital of Stomatology, Fujian Medical University, Fuzhou, China; 3 Department of Human Anatomy and Histology, and Embryology, School of Basic Medical Sciences, Fujian Medical University, Fuzhou, China; 4 Shanxi Medical University School and Hospital of Stomatology, Taiyuan, China

## Abstract

Oral squamous cell carcinoma (OSCC) remains one of the most aggressive malignancies of the oral epithelium, with limited therapeutic options effectively targeting both tumor growth and metastasis. Plant-derived nanovesicles have emerged as natural, biocompatible nanomedicines with potential applications in cancer therapy. Here, we systematically screened nanovesicles from ten medicinal plants and identified *Panax notoginseng*-derived nanovesicles (PnNVs) as the most potent inhibitors of OSCC. PnNVs exhibited favorable safety profiles and intrinsic tumor-homing ability, selectively accumulating in orthotopic tongue tumors. In vivo, they markedly suppressed primary tumor growth and lymphatic metastasis. Mechanistically, PnNVs disrupted redox homeostasis by inhibiting the p38–MAPK/NRF2 signaling pathway, thereby inducing ferroptosis, autophagy, and PANoptosis. In addition, PnNVs impaired cancer cell migration by modulating chemokine-associated signaling pathways critical for tumor dissemination. Multi-omic analyses further revealed synergistic contributions of RNA cargos and metabolite components to their multi-target anticancer efficacy. Collectively, our findings establish PnNVs as a natural, multifunctional therapeutic candidate with dual anti-proliferative and anti-metastatic activity, providing a promising preclinical strategy for OSCC treatment.

**Graphical Abstract:**

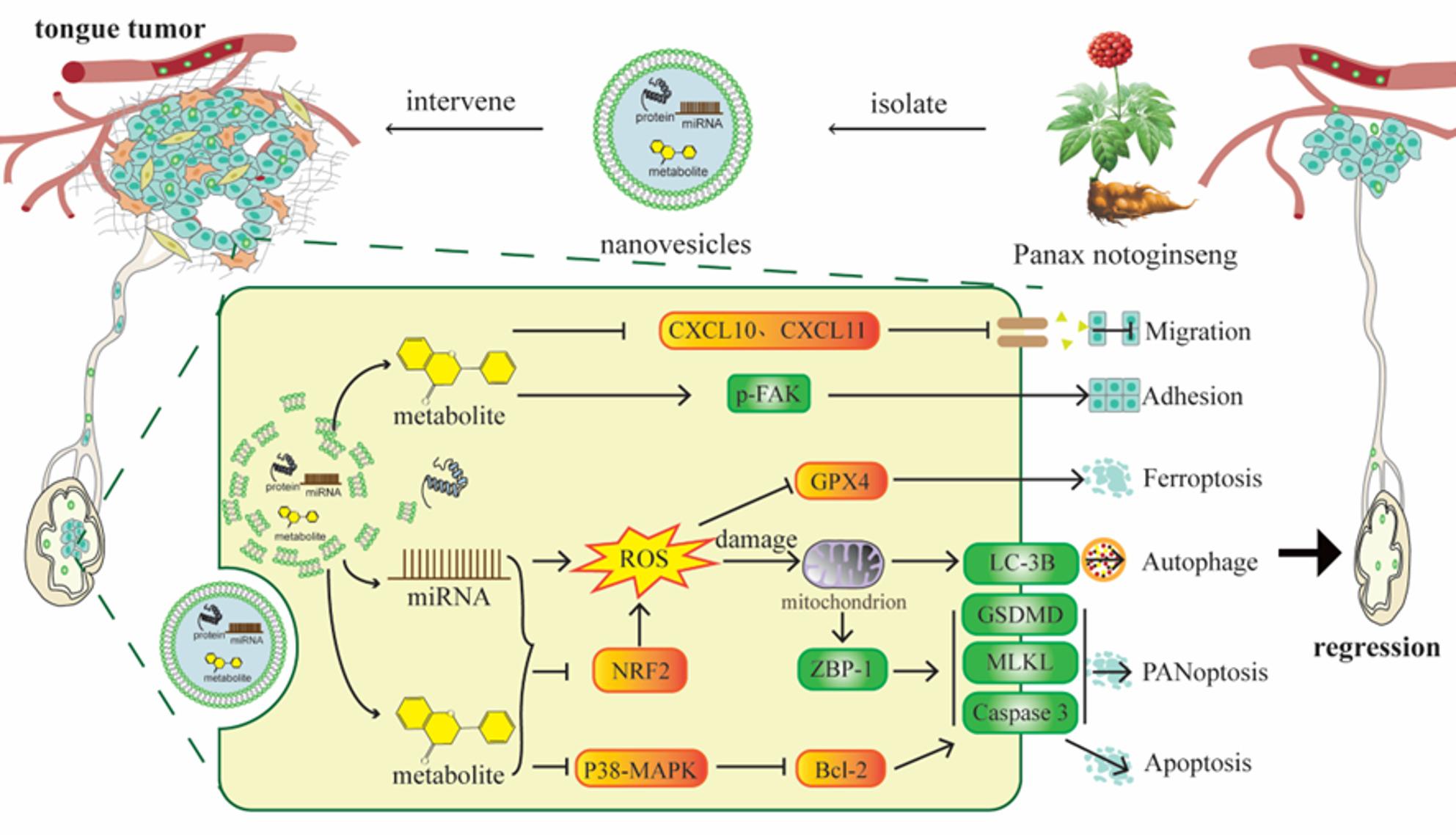

**Supplementary Information:**

The online version contains supplementary material available at 10.1186/s12951-026-04193-9.

## Highlights

- PnNVs act as potent natural inhibitors of oral squamous cell carcinoma.
- PnNVs target tongue tumors, suppressing growth and lymphatic metastasis in vivo.
- PnNVs trigger ferroptosis, autophagy, and PANoptosis via p38-MAPK/NRF2 signaling.
- PnNVs impair tumor migration via modulation of chemokine signaling pathways.
- Multi-omics reveal synergistic RNA and metabolite cargos driving anticancer efficacy.

**Supplementary Information:**

The online version contains supplementary material available at 10.1186/s12951-026-04193-9.

## Introduction

Oral squamous cell carcinoma (OSCC) is one of the most prevalent and lethal malignancies of the head and neck, accounting for over 90% of oral cancers [1, 2]. Despite advances in surgery, radiotherapy, and chemotherapy, prognosis remains poor, with five-year survival rates stagnating at ~50% [1, 3, 4]. Survival declines sharply with disease progression: approximately 80% for localized tumors, 60% for regional metastasis, and only 30% for distant metastasis, reflecting the heavy prognostic burden of cervical lymph node metastasis (LNM) involvement. Multiple evidence further demonstrates that LNM occurs in distinct cervical patterns in OSCC, emphasizing the clinical need to curb metastatic dissemination [5, 6]. However, Current treatment strategies, including combination chemotherapy and EGFR-targeted agents such as cetuximab, provide limited benefit, and treatment-related toxicities further compromise patient quality of life [7–12]. These challenges underscore the need for novel, low-toxicity therapeutics capable of simultaneously restraining OSCC growth and metastasis.

Natural products from medicinal plants have historically provided valuable anticancer agents, including paclitaxel and vincristine, which are already used in OSCC chemotherapy [13–15]. Compared with single-target therapies, plant-derived medicines often exert multi-targeted effects with fewer side effects. Several formulations have shown potential in suppressing OSCC proliferation and metastasis [16], and meta-analyses in other cancers, such as hepatocellular carcinoma and gastric cancer, demonstrate that herbal medicine or integrative regimens can reduce recurrence and improve patient survival [17–19]. These findings highlight the translational value of exploring plant-based therapeutics for OSCC.

Recently, plant-derived nanovesicles (PDNVs) have emerged as a novel class of natural nanomedicines. Isolated from edible and medicinal plants, PDNVs are bilayer nanostructures enriched with RNAs, proteins, lipids, and metabolites [20, 21]. Evidence indicates that nanovesicles may account for a major portion of the bioactivity in traditional herbal decoctions, suggesting they could represent key bioactive carriers underlying herbal efficacy [22, 23]. PDNVs display excellent biocompatibility and intrinsic biological activities, and accumulating studies have reported anticancer effects in diverse contexts [24–26]. For example, ginseng-derived nanovesicles have been shown to enhance antitumor immunity, bitter melon-derived nanovesicles to alleviate OSCC chemoresistance, and tea flower-derived nanovesicles to inhibit breast cancer metastasis through microbiome modulation [27–30].

Despite these advances, the roles of PDNVs in OSCC remain largely unexplored. Whether specific PDNVs can simultaneously inhibit both OSCC proliferation and metastasis has not been investigated. To explore this possibility, we systematically screened nanovesicles from ten medicinal plants and identified *Panax notoginseng*-derived nanovesicles (PnNVs) as the most effective candidates. We further evaluated their therapeutic efficacy in orthotopic OSCC models and dissected their molecular mechanisms through integrated transcriptomic and metabolomic analyses. This study demonstrates the dual anti-proliferative and anti-metastatic potential of PnNVs and provides a foundation for developing natural nanomedicines for OSCC therapy.

## Materials and methods

### Cell culture

The cell lines used in this study included LN4 cells (established in our lab through four rounds of in vivo selection from Cal-27 cells and verified by STR profiling [31]), SCC7 mouse squamous cell carcinoma cells (Yimo Biotech, China), NIH3T3 fibroblasts (Haixing Biotech), HEK293T cells (Cyagen OriCell), human umbilical vein endothelial cells (HUVECs, iCell Bioscience) , and lymphatic endothelial cells (Punosai Biotech). All cell types were maintained in high-glucose DMEM supplemented with 10% FBS, and endothelial cell medium (ECM) for endothelial cells. Cells were cultured at 37 °C with 5% CO₂, subcultured at ~ 80% confluence, and cryopreserved in 10% DMSO-containing medium at − 80 °C or in liquid nitrogen.

### Isolation and characterization of PDNVs

Ten medicinal plants were selected based on clinical relevance and geographical representation, and sourced from certified farms across China to ensure batch consistency: *Hedyotis diffusa* [32], *Lobelia chinensis* [33], and *Scutellaria barbata* [34] (Jieyang, Guangdong); *Gynostemma pentaphyllum* [35] (Pingdingshan, Henan); *Fritillaria* [36] (Enshi, Hubei); *Panax notoginseng* [37] (Wenshan, Yunnan); *Rehmannia glutinosa* [38] (Jiaozuo, Henan); *Polygonatum* [39] (Honghe, Yunnan); *Cirsium japonicum* [40] (Fuzhou, Fujian); and *Ficus carica L.* [41] (Chengdu, Sichuan).

Plant materials were rinsed with tap water followed by sterile distilled water. Tissues were harvested as follows: entire herbs for *Hedyotis diffusa*, *Lobelia chinensis*, and *Scutellaria barbata*; stems and leaves for *Gynostemma pentaphyllum*; roots/rhizomes for *Panax notoginseng*, *Fritillaria*, *Rehmannia glutinosa*, and *Cirsium japonicum*. Samples were homogenized in pre-chilled PBS (1:1, w/v) using a high-speed blender, followed by sequential centrifugation at 2,000 × g (10 min), 5,000 × g (20 min), and 10,000 × g (30 min) at 4 ℃ to remove debris. The supernatant was ultracentrifuged at 100,000 × g for 30 min to pellet nanovesicles, which were then washed with PBS and filtered through 0.45 μm and 0.22 μm membranes.

Purified PDNVs were either stored at 4 ℃ (< 1 week) or snap-frozen in liquid nitrogen and preserved at –80 ℃ for long-term use. Vesicle protein content was quantified using a BCA assay. Morphology was examined via transmission electron microscopy (TEM), while particle size and zeta potential were analyzed using dynamic light scattering (DLS).

### Cell proliferation assay

Cells were seeded into 96-well plates and treated with various concentrations of different PDNVs for 24 or 48 h. Cell viability was measured using the CCK-8 assay (10 μL reagent + 90 μL serum-free medium/well, incubated for 1 h). Absorbance at 450 nm was read using a microplate reader. Cell viability (%) was calculated as:

Cell viability (%) = ((OD_treatment − OD_blank)/(OD_control − OD_blank)) × 100%.

### Wound healing assay

Cells were seeded in 12-well plates to ~ 90% confluence. A cross-shaped scratch was made using a 200 μL pipette tip, followed by PBS washing and treatment with PDNVs (25, 50, 100 μg/mL). Images were acquired at 0 h and again when the wound in the control group had closed. Wound area was quantified using ImageJ.

Wound healing rate (%) = ((Area_0h − Area_healing)/Area_0h) × 100%.

### Construction of dual-luciferase labeled LN4 stable cell line

A dual-luciferase plasmid was cloned, sequence-verified, and packaged into lentivirus in HEK293T cells by co-transfection with psPAX2 and pMD2.G. Viral supernatants were collected after 48 h, filtered (0.45 μm), and used to transduce LN4 cells in the presence of polybrene (40 μg/mL). Stable clones were selected using puromycin (2 μg/mL) and validated by flow cytometry and bioluminescence imaging.

### Cellular uptake of *panax notoginseng*-derived nanovesicles

SCC7 and LN4 cells were incubated with DiO-labeled PnNVs (4 mg/mL) for 24 h. After fixation and staining with phalloidin and DAPI, uptake was visualized by confocal microscopy. Parallel uptake quantification at 1, 12, and 24 h was conducted by flow cytometry based on FITC signal intensity.

### In vivo tongue cancer model establishment and therapeutic intervention

All animal experiments were approved by the Ethics Committee of Fujian Medical University (Approval No. IACUC FJMU 2023–0238) and performed under specific pathogen-free (SPF) conditions. Male BALB/c-nu nude mice (6–8 weeks old, Guangdong Yaokang Biotechnology, China) were acclimatized for one week prior to tumor inoculation. Bioluminescent LN4 OSCC cells (2 × 10^5^ in 15 μL serum-free medium) were injected into the lateral border of the tongue under isoflurane anesthesia. Mice were randomly assigned (n = 10/group) based on body weight and baseline fluorescence.

Tumor growth and cervical lymph node metastasis were monitored by bioluminescence imaging on days 7 and 28 post-inoculation. To compensate for tumor-induced feeding impairment, supplemental whole milk was provided in addition to standard chow. D-luciferin (150 mg/kg) was injected intraperitoneally 10 min before imaging. Fluorescence intensities within regions of interest (ROI) were quantified to assess primary tumor burden and metastatic spread. PnNVs were administered intraperitoneally at (200 μL, 2 mg/mL) every other day for three weeks. At study endpoint, mice were euthanized for collection of blood, tumors, lymph nodes, and major organs for biochemical and histopathological analyses. Blood was collected to analyze serum alanine aminotransferase (ALT) and creatinine levels.

### In vivo biodistribution of PnNVs

To assess biodistribution, orthotopic tongue tumor models were established using LN4 cells without fluorescent reporters to avoid signal interference. Two weeks post-inoculation, mice bearing visible tongue tumors received intraperitoneal injections of DIR -labeled PnNVs (500 μL, 2 mg/mL). Unbound dye was removed by ultracentrifugation and size-exclusion chromatography.

At 12, 24, and 48 h post-injection, mice were euthanized and major organs—including the heart, liver, spleen, lungs, kidneys, and tumor-bearing tongues—were collected. Tissues were rinsed with cold PBS and gently blotted dry. Ex vivo fluorescence imaging was performed using a small animal imaging system, and fluorescence intensities were quantified within ROIs defined for each organ using the accompanying analysis software.

### Tissue processing and H&E staining

At the experimental endpoint, organs, tongue tumors, and cervical lymph nodes were harvested, rinsed with PBS, and fixed in 4% paraformaldehyde for 48 h. After ethanol dehydration and butanol clearing, tissues were embedded in paraffin and sectioned (3–5 μm). For H&E staining, sections were deparaffinized, rehydrated, stained with hematoxylin and eosin, dehydrated, and mounted with resin before microscopic examination.

#### EdU incorporation assay

Cells were seeded in 24-well plates and treated with PnNVs for 24 h. EdU reagent (1:1000) was added for 1 h, followed by fixation, permeabilization, Click-iT™ reaction, and Hoechst counterstaining. Fluorescent signals were observed using confocal microscopy.

#### Live/dead cell staining assay

Cells were treated with PnNVs for 24 h, collected, and stained with calcein-AM (live cells) and propidium iodide (dead cells). Fluorescence signals were captured by confocal microscopy.

#### Dead cell PI staining assay

Cells were treated with intact PnNVs, depleted vesicles, or isolated fractions for 24 h. PI (1:1000) was added directly to the culture medium and incubated in the dark. Both suspended and adherent cells were collected and analyzed by flow cytometry.

#### TUNEL staining for apoptosis detection

Tissue sections were dewaxed, washed with PBS, and permeabilized with Proteinase K (100 μL per section, 37 ℃, 30 min), then washed thoroughly with PBS. Positive controls were treated with DNase I (100 μL, 37 ℃, 30 min). For TUNEL reaction, slides were incubated with TdT reaction buffer containing biotin-11-dUTP and TdT enzyme (50 μL per sample) at 37 ℃ for 60 min in a humidified chamber protected from light; negative controls lacked TdT. After washing, slides were incubated with streptavidin-TRITC (50 μL per section) at 37 ℃ for 30 min in the dark. Nuclei were counterstained with DAPI-containing antifade medium. Fluorescence was visualized and recorded using confocal microscopy (excitation 543 nm; emission 571 nm), with prompt image capture to avoid signal loss.

#### Immunohistochemistry (IHC)

Paraffin-embedded tissue sections were deparaffinized and rehydrated through graded ethanol. Antigen retrieval was conducted by heating sections in citrate buffer (1:1000) under pressure, followed by cooling and blocking of endogenous peroxidase with 3% hydrogen peroxide (H_2_O_2_). Sections were then blocked with 50 μL normal goat serum for 10 min.

The sections were incubated with the following primary antibodies: anti-Ki-67 (1:600, Abcam, Cat# ab15580), anti-ZBP1 (1:500, Proteintech, Cat# 13285–1-AP), anti-phospho-MLKL (p-MLKL) (1:50, Zen-bio, R382136), anti-Cleaved Caspase-3 (1:800, Proteintech, Cat# 25128–1-AP), anti-GPX4 (1:100, Cell Signaling Technology, 59735), anti-Cleaved GSDMD (1:500, Cell Signaling Technology, 36425), and anti-Cleaved GSDME (1:300, ABclonal, A26197). Primary antibody incubation was performed at 37 °C for 1 h or overnight at 4 °C.

After washing with PBS, sections were incubated with horseradish peroxidase (HRP)-conjugated secondary antibody for 20 min at room temperature. Immunoreactivity was visualized using 3,3′-diaminobenzidine (DAB), followed by hematoxylin counterstaining. Slides were subsequently dehydrated, cleared, and mounted. Images were acquired using a phase-contrast inverted microscope.

#### RNA sequencing and transcriptomic analysis

LN4 cells were treated with PnNVs for 48 h. Total RNA was extracted using TRIzol and sent to OE Biotech (Shanghai, China) for library preparation and sequencing on the Illumina platform. Differentially expressed genes (DEGs) were identified and subjected to GO and KEGG pathway enrichment analysis using standard bioinformatics pipelines.

#### Apoptosis assay by PI/Annexin V staining

After 24 h of PnNVs treatment, floating and adherent cells were harvested, stained with Annexin V and PI, and analyzed by flow cytometry. Adherent cells were gently trypsinized and incubated in serum-free medium before staining to minimize false-positive signals.

#### Western blot analysis

Cells were seeded in 6-well plates at 1 × 10^6^ cells/well and treated with PnNVs (25 or 50 μg/mL for proliferation assays; 12.5 or 25 μg/mL for migration assays) for 24 h. After treatment, cells were washed twice with ice-cold PBS and lysed in RIPA buffer containing protease and phosphatase inhibitors (1 mM PMSF, 1 mM Na₃VO₄, and a protease inhibitor cocktail). Lysates were sonicated on ice and centrifuged at 12,000 rpm for 10 min at 4 ℃. Protein concentration was measured using BCA assay.

Equal amounts of protein (1 mg/mL) were mixed with 5 × SDS loading buffer, boiled at 100 ℃ for 10 min, separated by 4–12% SDS-PAGE, and transferred onto PVDF membranes (400 mA, 40 min). Membranes were blocked with 3% BSA for 30 min at room temperature and incubated overnight at 4 ℃ with primary antibodies diluted 1:1500.

Primary antibodies included Caspase-3 (#9662), Cleaved-Caspase-3 (#9664), GAPDH (#5174), HSP90 (#4874), MAPK (#4695), phospho-MAPK (#4370), p38 (#9212), phospho-p38 MAPK (#4511), JNK (#9252), phospho-JNK (#4668), SNAIL (#3879), SLUG (#9585), CHD1 (#4351), CHD2 (#4170), Cleaved-GSDME (55879 T) , GPX4 (59735), (Cell Signaling Technology); GSDMD (SY-R24514), Cleaved-GSDMD (R40133), MLKL (R24989), phospho-MLKL (R382136), GSDME (R24108), TFRC (R383050), CCL5 (R382169), CXCL10 (R381735), CXCL11 (SY-R382999) (Zen Bioscience); ZBP-1 (13,285–1-AP), FAK (66,258–1-Ig), p-FAK (83,933–1-RR) (Proteintech).

Membranes were incubated with HRP-conjugated secondary antibodies (anti-rabbit IgG 1:3000 or anti-mouse IgG 1:5000) for 45 min at room temperature. After washing, signals were detected using ECL reagents (Thermo Fisher Scientific). Band intensities were quantified with ImageJ.

#### Quantitative real-time PCR (qRT-PCR)

Total RNA was extracted from cells treated with PnNVs for 24 h. RNA concentration was measured, and cDNA was synthesized using a two-step reverse transcription kit (Takara, Japan) following the manufacturer’s instructions: genomic DNA removal at 42 ℃ for 2 min, reverse transcription at 37 ℃ for 15 min, and enzyme inactivation at 85 ℃ for 5 s. cDNA was stored at −20 ℃ until use.

qPCR was performed using TB Green® Premix Ex Taq II (Takara) in 20 μL reactions containing 10 μL TB Green, 1 μL each primer (0.2 μM), 2 μL cDNA, and 7 μL nuclease-free water. Amplification was carried out on a Bio-Rad CFX96 system using GAPDH as the internal control. Relative gene expression was calculated by the 2^−ΔΔCt^ method.

Primer sequences are listed below:

**Table:** 

Gene	Forward Primer (5'–3')	Reverse Primer (5'–3')
CD14	GTACTCCCGCCTCAAGGAAC	CGCAAGCTGGAAAGTGCAAG
NGFR	TCATCCCTGTCTATTGCTCCAT	TGTTGGCTCCTTGCTTGTTC
DUSP9	GGAGGCCATTGAGTTCATTGAT	AGGTCATAGGCATCGTTGAGA
CXCL1	GGGAATTCACCCCAAGAACATC	GGATGCAGGATTGAGGCAAGC
CCL2	CAGCAAGTGTCCCAAAGAAGC	TCGGAGTTTGGGTTTGCTTG
CCL20	GCAAGCAACTTTGACTGCTG	TTGGATTTGCGCACACAGAC
CXCL10	GTGGCATTCAAGGAGTACCTC	TGATGGCCTTCGATTCTGGATT

#### JC-1 mitochondrial membrane potential assay

Mitochondrial membrane potential (ΔΨm) was assessed using JC-1 staining. LN4 cells (5 × 10^5^/well) were seeded in 6-well plates and divided into control, CCCP (50 μM, 20 min) positive control, and PnNVs-treated groups (24 h). After washing with PBS, cells were incubated with JC-1 working solution at 37 °C for 20 min. JC-1 fluorescence was visualized by laser scanning confocal microscopy. Red/green fluorescence ratios were quantified with flow cytometry and analyzed using FlowJo.

#### Intracellular reactive oxygen species (ROS) detection

Intracellular ROS levels were detected using the DCFH-DA fluorescent probe. Cells were seeded in 12- or 24-well plates and divided into control, H₂O₂-treated (1:1000, 30 min) positive control, and PnNVs-treated groups (12 h). After incubation, cells were washed and stained with DCFH-DA (1:1000 in serum-free medium) at 37 °C for 30 min. ROS signals were assessed using confocal microscopy and flow cytometry, and quantified with FlowJo.

#### Transwell migration assay

Cell migration was assessed using 24-well Transwell chambers with 8.0 μm pore size inserts (Corning, USA). Cells were resuspended in serum-free medium (600 μL, upper chamber), while the lower chamber contained 800 μL complete medium supplemented with PnNVs. SCC7 cells were incubated for 24 h and LN4 cells for 48 h at 37 ℃. Non-migrated cells on the upper membrane surface were gently removed with a cotton swab. Migrated cells on the lower surface were fixed with 4% paraformaldehyde for 10 min and stained with 0.1% crystal violet for 5 min. Images were acquired with a microscope.

#### Matrigel invasion assay

Matrigel-coated Transwell chambers (Corning, USA) were coated with Matrigel, activated using poly-L-lysine, and equilibrated in serum-free medium for 2 h at 37 ℃. SCC7 (3 × 10^4^) and LN4 (6 × 10^4^) cells were resuspended in 600 μL serum-free medium and seeded into the upper chambers. The lower chambers contained 800 μL complete medium with PnNVs. After 48 h (SCC7) or 72 h (LN4) incubation, non-invaded cells on the upper membrane surface were removed. Invaded cells were fixed with 4% paraformaldehyde and stained with 0.1% crystal violet for 5 min. Images were captured with a microscope.

#### Cell adhesion assay

LN4 cells labeled with dual-luciferase and pretreated with PnNVs were harvested and resuspended. Confluent monolayers of HUVECs, lymphatic endothelial cells, or LN4 cells (2 × 10^5^ cells/well) were prepared in 48-well plates. Pretreated LN4 cells were added and allowed to adhere for 15 min at 37 ℃. Non-adherent cells were gently washed off. Adherent cells were fixed with 4% paraformaldehyde and imaged for quantification.

#### Component characterization and selective depletion in PnNVs

To evaluate the functional contribution of individual biomolecules in PnNVs, total RNA, proteins, and lipids were selectively extracted, characterized, and enzymatically removed using standard protocols.

**RNA** was extracted using TRIzol reagent following ultracentrifugation of freshly isolated PnNVs. Purity and concentration were assessed with a NanoDrop spectrophotometer, and integrity confirmed via agarose gel electrophoresis. For RNA depletion, nanovesicles (1 mg protein equivalent) were treated with RNase A (10 mg/mL, 37 ℃, 30 min), followed by ultracentrifugation and PBS resuspension. Post-treatment RNA removal was validated through gel electrophoresis.

**Proteins** were extracted using RIPA buffer and analyzed by SDS-PAGE with Coomassie staining. Proteinase K (20 mg/mL, 37 ℃, 30 min) was used for depletion, followed by ultracentrifugation and dialysis to remove residual enzyme. Successful protein removal was confirmed through repeat SDS-PAGE.

**Lipids** were extracted using chloroform: methanol: PnNVs (8:4:3, v/v/v), with the organic phase evaporated at 65 ℃. Resuspended lipids were subjected to TLC (chloroform: methanol: acetic acid, 190:9:1) and visualized with 8% CuSO₄ followed by heating. Bands were documented with a gel imaging system.

These workflows enabled reliable profiling and functional depletion of PnNVs components, allowing downstream evaluation of their individual roles in tumor cell phenotypes.

#### miRNA sequencing analysis of PnNVs

miRNA sequencing was performed on three independent PnNVs samples (Wuhan Jinkairui Bioengineering Co., Ltd.). Total miRNA was extracted using the miRNeasy Mini Kit, followed by cDNA synthesis and library construction with adapters containing a Qiagen-specific sequence, a 12-nt UMI, and Illumina adapter sequences. High-throughput sequencing was conducted, and reads were aligned for miRNA identification and target prediction. GO and KEGG pathway enrichment analyses were performed.

#### Lipid metabolomics

Lipid extraction and analysis were performed on PnNVs. Samples were vortexed with 400 μL methanol for 1 min, centrifuged (12,000 rpm, 10 min, 4 °C), and the supernatant was collected, concentrated, and dried. Residues were reconstituted in 150 μL of 80% methanol containing 2-chloro-L-phenylalanine (4 ppm), filtered (0.22 μm), and transferred to vials. Samples were shipped on dry ice to Suzhou Panomics Biomedical Technology Co., Ltd. for quality control, mass spectrometry, and lipidomic data analysis.

#### Statistics analyses

Data are expressed as mean ± standard deviation. For comparisons between two groups, either Student’s t-test or Mann–Whitney U test was used based on data distribution. For multiple group comparisons, homogeneity of variance was first assessed; One-Way Analysis of Variance (ANOVA) was applied for data meeting homogeneity, while the Kruskal–Wallis H test was used when variances were unequal. A p-value less than 0.05 was considered statistically significant.

## Results

### Screening identifies PnNVs as the potent inhibitors of OSCC

**Fig. 1:**
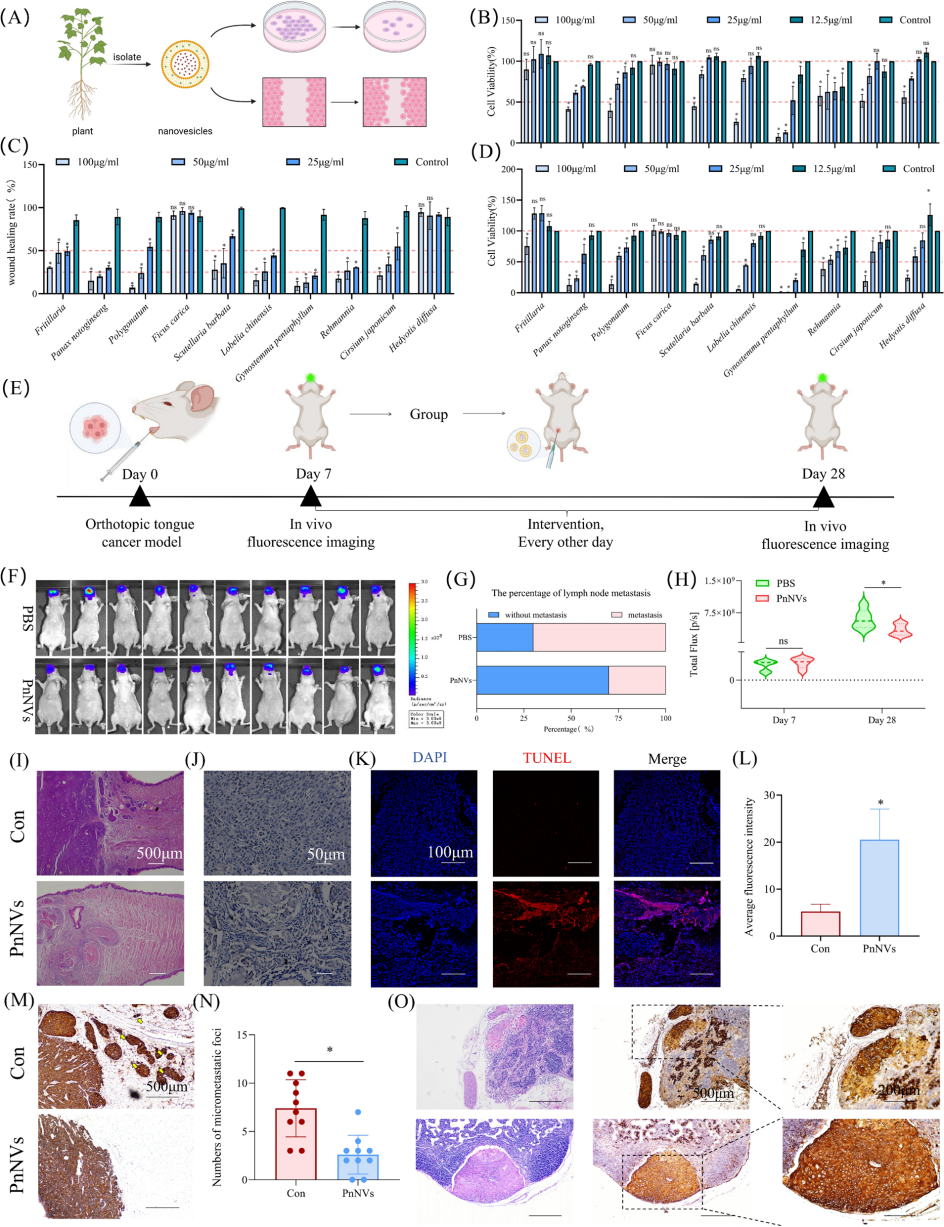
Screening of PDNVs for anti-OSCC activity. (**A**) Schematic diagram illustrating the intervention in tumor cells by PDNVs. (**B, D**) Cell viability of LN4 cells treated with PDNVs for 48 h (**B**) and 72 h (**D**), measured by CCK-8 assay. (**C**) Wound healing assay of LN4 cells following treatment with PDNVs. (**E**) Experimental workflow of the orthotopic tongue tumor model and intraperitoneal administration of selected nanovesicles. (**F–H**) Bioluminescence imaging (**F**), cervical lymph node metastasis incidence (**G**), and primary tumor fluorescence intensity (**H**) following treatment with PnNVs. (**I**) H&E staining shows that tongue tumor volume is markedly reduced after PnNVs treatment, (**J**) accompanied by decreased proliferative activity as indicated by Ki67 immunohistochemistry. (**K, L**) TUNEL staining reveals elevated apoptosis in tumors from the PnNVs-treated group, evidenced by stronger red fluorescence signals and corresponding quantitative differences. (**M**) Representative histological sections and (**N**) quantitative analysis demonstrate a significant reduction in microscopic metastatic foci within tongue tissues following PnNVs administration. (**O**) H&E and PanCK staining further confirm a marked reduction in lymph-node and peri-nodal metastatic lesions after PnNVs intervention. (Abbreviations: PDNVs, plant-derived nanovesicles; OSCC, oral squamous cell carcinoma; CCK-8, Cell Counting Kit-8; PnNVs, *Panax notoginseng*-derived nanovesicles)

Nanovesicles isolated from ten medicinal plants traditionally associated with anti-cancer activity were first evaluated for their ability to inhibit OSCC progression. In vitro assays showed that several PDNVs, including those from *Panax notoginseng* (PnNVs), *Gynostemma pentaphyllum* (GpNVs), and *Rehmannia glutinosa* (R-NVs), exerted comparable inhibitory effects on OSCC cell proliferation and migration, without a clear lead candidate (Fig. 1A-D).

To further determine their therapeutic potential, we next tested representative PDNVs in an orthotopic tongue cancer model (Fig. 1E). Strikingly, PnNVs exhibited the most pronounced anti-tumor activity in vivo, significantly reducing primary tumor burden and cervical lymph node metastasis (Fig. 1F-H), whereas the other PDNVs showed only partial or minimal effects (Figure S1). These findings identified PnNVs as the *optimal candidate* for downstream mechanistic studies.

We further examined tumor progression in vivo following PnNVs treatment. Histological analysis showed that the tongue tumors in the PnNVs-treated group were markedly smaller than those in the PBS group (Fig. 1I). In parallel, immunohistochemical staining revealed a substantial reduction in Ki67 expression after PnNVs administration (Fig. 1J). Consistently, TUNEL staining demonstrated a stronger red fluorescence signal in PnNVs-treated tumors (Fig. 1K), accompanied by a statistically significant increase in TUNEL-positive cells (Fig. 1L).

In addition to the indicators of tumor growth, we focused on the presence of microscopic metastatic lesions. Notably, the number of micro-metastatic foci in the tongue tissue was significantly reduced in the treatment group (Fig. 1M and 1 N), suggesting that PnNVs may suppress the metastatic potential of OSCC. Bioluminescence imaging further showed a marked decrease in cervical lymph node signals after treatment, which was consistent with the findings observed upon dissection of cervical tissues. Moreover, PanCK and H&E staining provided additional evidence for the pronounced reduction of lymph-node and peri-nodal metastatic lesions following PnNVs intervention (Fig. 1O).

### Physicochemical characterization, biodistribution, and biosafety of PnNVs

**Fig. 2:**
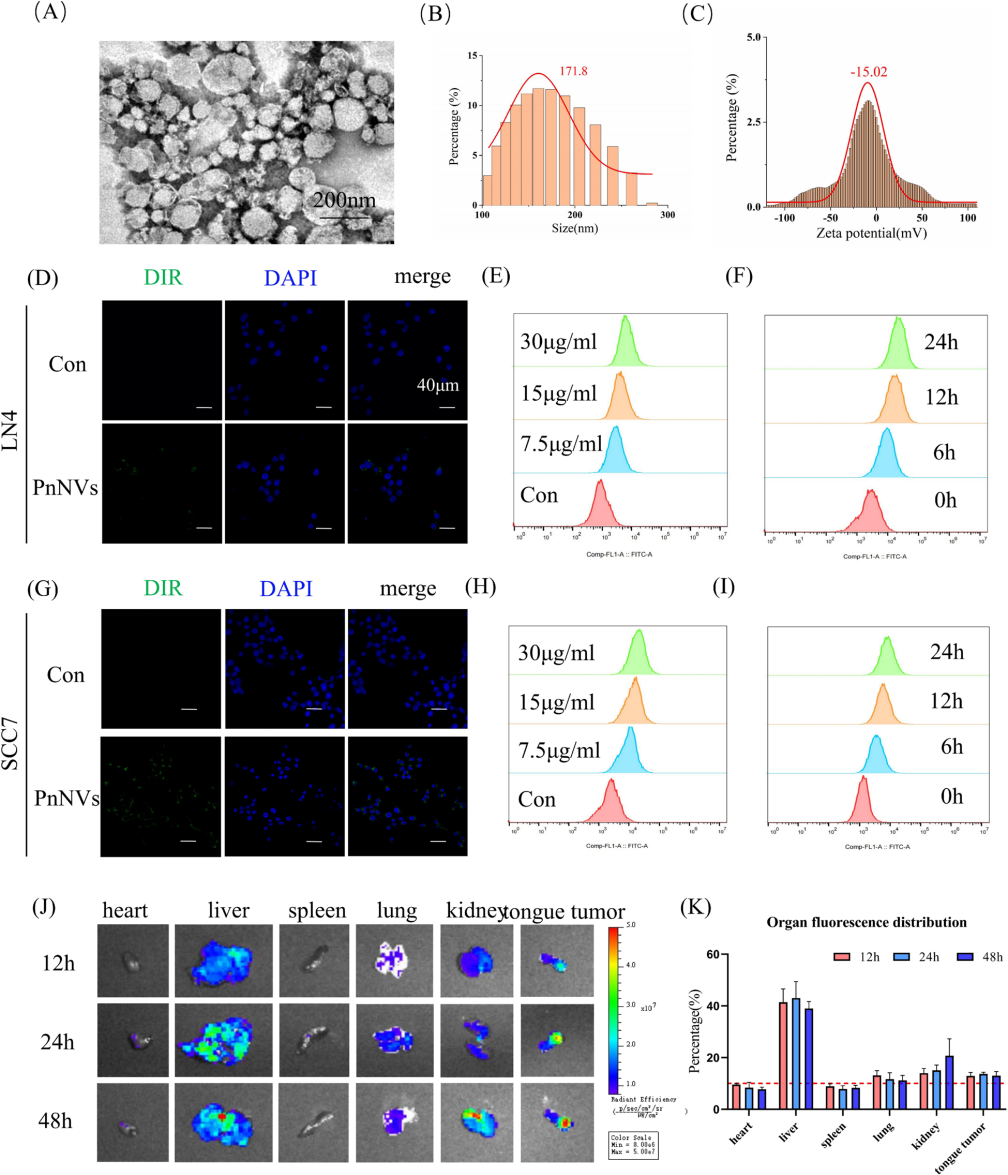
Physicochemical characterization, cellular uptake, and biodistribution of PnNVs. (**A–C**) Physicochemical characterization of Panax notoginseng-derived nanovesicles (PnNVs), including (**A**) transmission electron microscopy (TEM) imaging, (**B**) particle size distribution (nm), and (**C**) zeta potential profile (mV). (**D–F**) Uptake of DiO-labeled PnNVs by LN4 cells: (**D**) representative confocal image showing intracellular accumulation (green) with nuclei counterstained by DAPI (blue), (**E**) dose-dependent uptake after 24 h, and (**F**) time-dependent uptake over 0–24 h. (**G–I**) Uptake of DiO-labeled PnNVs by SCC7 cells: (**G**) representative confocal image, (**H**) dose-dependent uptake after 24 h, and (**I**) time-course uptake over 0–24 h. (**J–K**) In vivo biodistribution of DiR-labeled PnNVs in orthotopic OSCC models: (**J**) ex vivo fluorescence imaging of major organs and tongue tumors at 12, 24, and 48 h post-injection, and (**K**) quantification of fluorescence intensity across organs.(Abbreviations: TEM, transmission electron microscopy; DiO/DiR, lipophilic fluorescent dyes)

Subsequently, we characterized the physicochemical properties of PnNVs, confirming their nanoscale size, stable surface charge, and typical bilayer vesicular morphology (Fig. 2A–C). Fluorescent tracing in multiple OSCC cell lines revealed that PnNVs were internalized in a time-dependent manner, indicating efficient and progressive cellular uptake (Fig. 2D–I). In vivo, systemic administration resulted in the natural accumulation of PnNVs within tongue tissues, with clear enrichment at the tumor site (Fig. 2J–K).

PnNVs also displayed a favorable safety profile. They did not affect the viability of normal fibroblasts in vitro, and repeated administration in mice caused no weight loss, biochemical abnormalities, or histological damage in major organs (Figure S2). Collectively, these results highlight PnNVs as the most potent and safe candidate for subsequent mechanistic studies.

### PnNVs suppress malignant proliferation of OSCC cells by inducing ferroptosis and PANoptosis

**Fig. 3:**
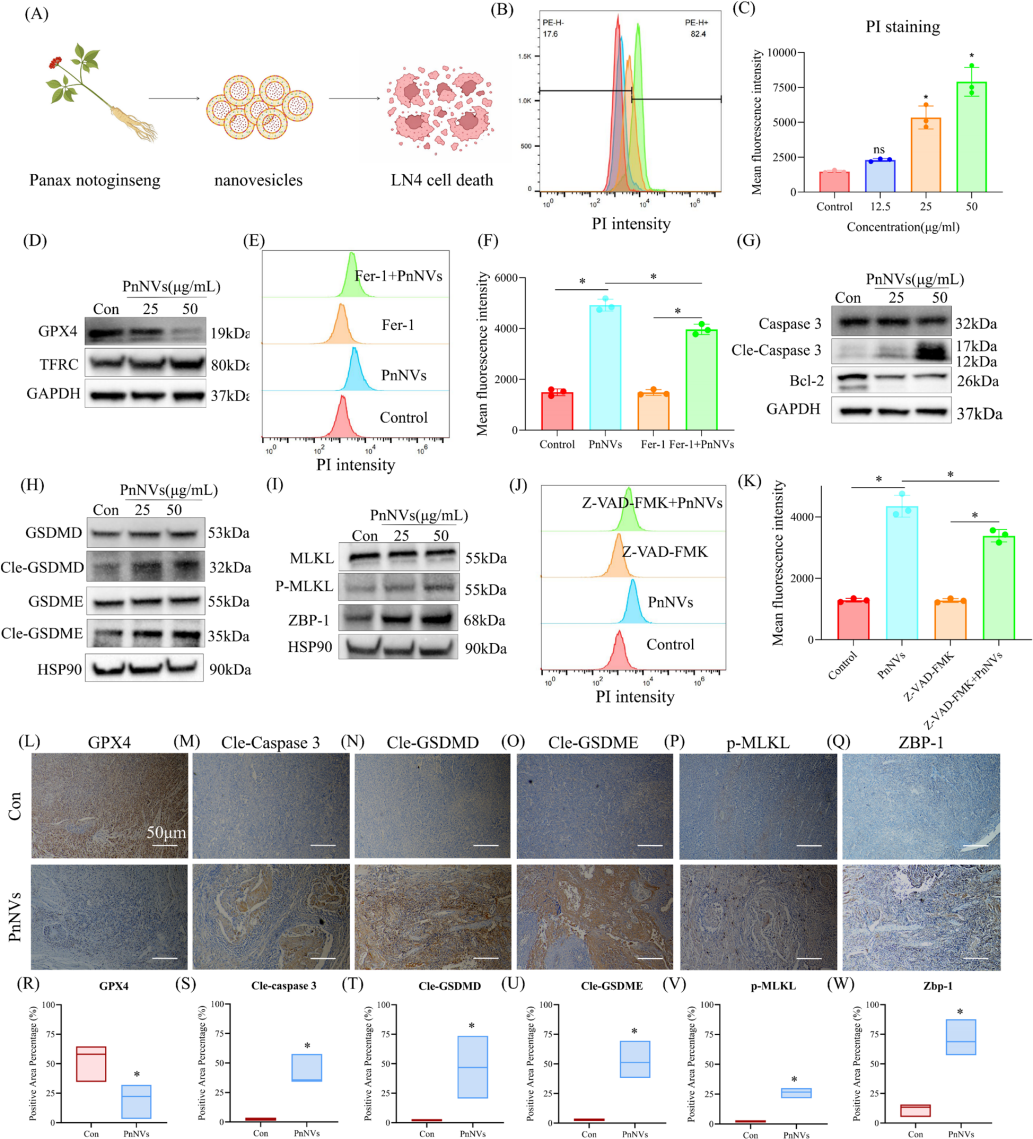
PnNVs suppress OSCC cell proliferation and induce multiple forms of cell death. (**A**) Schematic overview of the experimental strategy used to evaluate the effects of PnNVs on LN4 cell proliferation and death. (**B-C**) Flow cytometry analysis showing PI fluorescence intensity shift (**B**) and quantification (**C**) in LN4 cells after treatment with various concentrations of PnNVs. (**D**) Western blot analysis showing decreased GPX4 and increased TFRC expression after PnNVs treatment. Flow cytometry analysis showing PI fluorescence intensity shift (**E**) and quantification (**F**) in LN4 cells after treatment with PBS, PnNVs, Ferrostatin-1, and the group pretreated with Ferrostatin-1 followed by PnNVs. (**G**) Western blot analysis showing increased cleaved Caspase-3 and decreased Bcl-2 expression after PnNVs treatment. (**H**) Western blot analysis showing decreased Cle-GSDMD and Cle-GSDME expression after PnNVs treatment. (**I**) Western blot analysis showing increased phosphorylation of MLKL and elevated ZBP1 expression after PnNVs treatment. Flow cytometry analysis showing PI fluorescence intensity shift (**J**) and quantification (**K**) in LN4 cells after treatment with PBS, PnNVs, Z-VAD-FMK, and the group pretreated with Z-VAD-FMK followed by PnNVs. Immunohistochemical (IHC) staining of cell death–related markers in tongue tumor tissues from PBS- and PnNVs-treated mice. Representative images and statistics of positive areas show reduced (**L, R**) GPX4 expression and increased expression of (**M, S**) cleaved Caspase-3, (**N, T**) cleaved GSDMD, (**O, U**) cleaved GSDME, (**P, V**) phosphorylated MLKL (p-MLKL), and (**Q, W**) ZBP1 in PnNVs-treated tumors compared with PBS-treated controls. Scale bar, 50 μm. (Abbreviations: EdU, 5-ethynyl-2′-deoxyuridine; PI, propidium iodide; H&E, hematoxylin and eosin; TUNEL, terminal deoxynucleotidyl transferase dUTP nick end labeling; GPX4, glutathione peroxidase 4; TFRC, transferrin receptor; Bcl-2, B-cell lymphoma 2; GSDMD, gasdermin D; GSDME, Gasdermin-E; MLKL, mixed lineage kinase domain-like protein; ZBP1, Z-DNA binding protein 1)

To further assess the inhibitory effects of PnNVs on OSCC (Fig. 3A), we examined their impact on LN4 cell proliferation using EdU incorporation assays. At 24 h post-treatment, EdU staining revealed a significant reduction in DNA synthesis in PnNVs-treated LN4 cells (Figure S3A). A comparable antiproliferative effect was observed in SCC7 cells, known for their higher proliferative activity (Figure S3C-D). Notably, morphological features of cell death were observed in PnNVs-treated cultures, prompting further investigation. Live/dead staining showed a substantial increase in dead (red) cells within both LN4 and SCC7 populations (Figure S3B and S2E), and flow cytometry analysis confirmed increased PI-positive populations and elevated red fluorescence intensity (Fig. 3B-C, S3F-G).

To elucidate the mechanisms underlying PnNVs-induced cell death, we first performed transcriptomic profiling analysis on PnNVs-treated LN4 cells. Ferroptosis emerged as one of the most significantly enriched pathways (Figure S3H). Among ferroptosis-related genes, the key suppressor glutathione peroxidase 4 (GPX4) [42] was markedly downregulated, and transferrin receptor (TFRC) was increased (Fig. 3D). Moreover, pretreatment with the ferroptosis inhibitor Ferrostatin-1 partially reversed the PnNVs-induced cytotoxic effect (Fig. 3E–F), further substantiating the involvement of ferroptosis.

In addition to ferroptosis, transcriptomic and GSEA analyses revealed significant enrichment of apoptosis (hsa04210) (Figure S3I), the NOD-like receptor signaling pathway(hsa04621) (Figure S3J), and necroptosis (hsa04217) (Figure S3K), suggesting the concomitant activation of PANoptosis-related processes. At the protein level, cleaved Caspase-3 was elevated while total Caspase-3 remained unchanged, and anti-apoptotic B-cell lymphoma 2 (Bcl-2) was significantly downregulated, supporting apoptosis activation (Fig. 3G). For pyroptosis, both cleaved Gasdermin D (GSDMD) [43] and cleaved GSDME [44, 45] were upregulated (Fig. 3H). In parallel, the necroptosis execution marker phosphorylated mixed lineage kinase domain-like protein (p-MLKL) [46], was also significantly increased (Fig. 3I). Strikingly, expression of Z-DNA binding protein 1 (ZBP1), a central regulator of PANoptosis [47], was markedly upregulated (Fig. 3I), suggesting concurrent activation of apoptosis, necroptosis, and pyroptosis. Furthermore, pretreatment with the pan-caspase inhibitor Z-VAD-FMK attenuated the PnNVs-induced cell death phenotype (Fig. 3J–K), confirming the involvement of caspase-dependent programmed cell death pathways.

Subsequently, immunohistochemical staining was performed on tongue tumor tissues following PnNVs treatment to evaluate multiple cell death–related markers. Compared with PBS-treated control tumors, PnNVs-treated tumor tissues exhibited a marked reduction in GPX4-positive staining (Fig. 3L and 3R), whereas the expression of cleaved Caspase-3 (Fig. 3M and 3S), cleaved GSDMD (Fig. 3N and 3 T), cleaved GSDME (Fig. 3O and 3U), p-MLKL (Fig. 3P and 3 V), and ZBP1 (Fig. 3Q and 3 W) was significantly increased, as evidenced by enhanced brown immunoreactive signals. These results indicate that PnNVs treatment is associated with the activation of multiple regulated cell death pathways in vivo. Collectively, these results demonstrate that PnNVs induce ferroptosis and PANoptosis -associated cell death programs, which may collectively contribute to the suppression of OSCC cell proliferation and malignancy.

### PnNVs suppress MAPK and NRF2 signaling pathways to trigger ROS-dependent autophagic cell death in OSCC

**Fig. 4:**
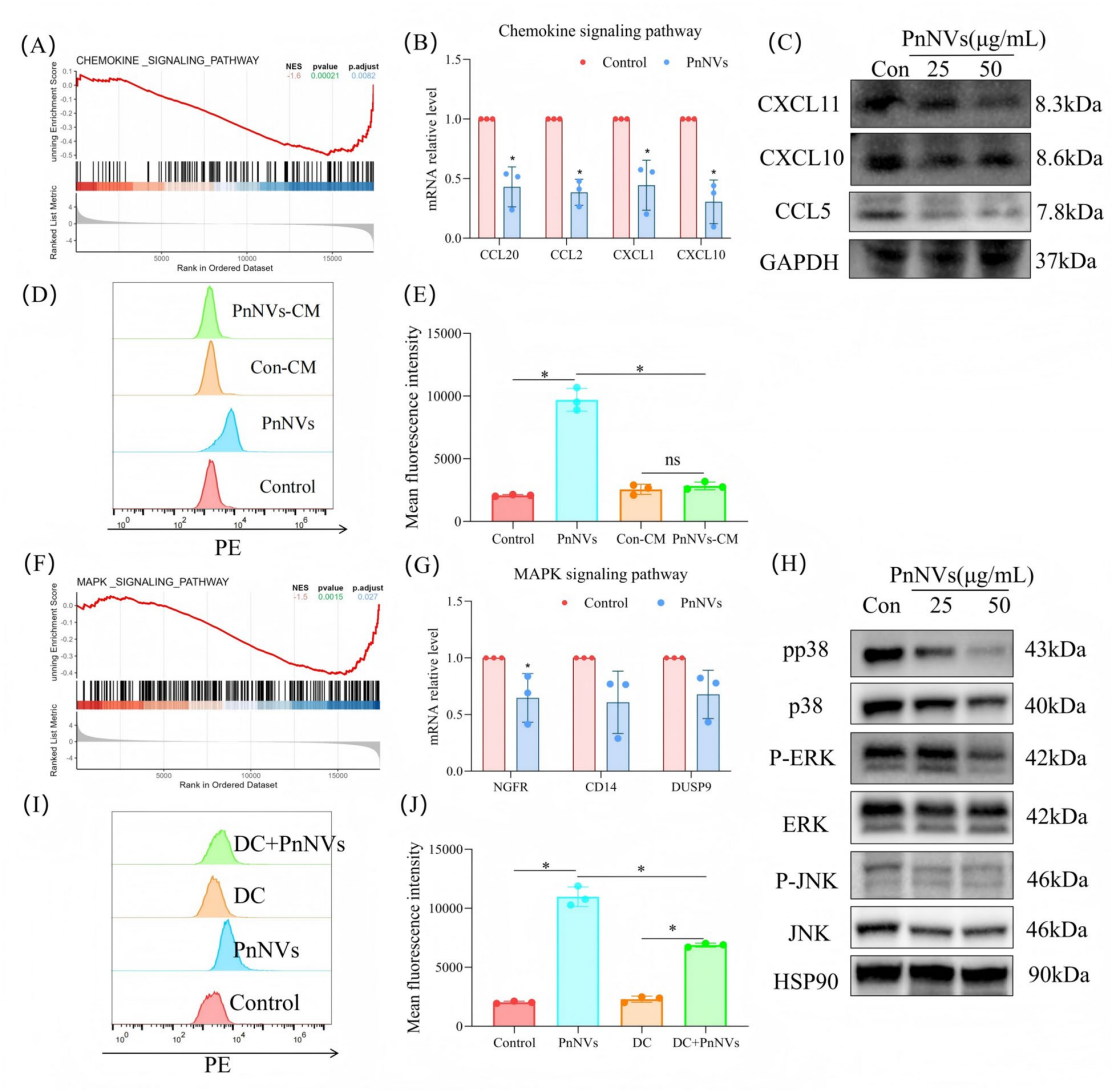
PnNVs inhibit OSCC cell proliferation primarily via MAPK suppression rather than chemokine signaling. (**A**) GSEA showing significant enrichment of the chemokine signaling pathway in transcriptomic data from PnNVs-treated LN4 cells. (**B**) RT-qPCR validation of chemokine-related gene expression changes (e.g., CCL5, CXCL10). (**C**) Western blot confirming reduced expression of CCL5 and CXCL10 proteins. (**D–E**) PI staining peak distribution (**D**) and PE channel fluorescence intensity (**E**) showing that conditioned medium from PnNVs-treated cells does not reproduce the cytotoxic effect of direct PnNV exposure. (**F**) GSEA indicating significant suppression of the MAPK signaling pathway. (**G**) RT-qPCR of MAPK-related gene expression changes. (**H**) Western blot showing decreased phosphorylation of p-p38 and p-ERK in PnNVs-treated cells. (**I–J**) Rescue experiments showing that MAPK activation with DC partially restores cell viability, as measured by PI peak distribution (**I**) and PE fluorescence intensity (**J**). (Abbreviations: PnNVs, *Panax notoginseng*-derived nanovesicles; OSCC, oral squamous cell carcinoma; GSEA, Gene Set Enrichment Analysis; RT-qPCR, reverse transcription quantitative PCR; CCL5, C–C motif chemokine ligand 5; CXCL10, C-X-C motif chemokine ligand 10; PI, propidium iodide; PE, phycoerythrin channel; MAPK, mitogen-activated protein kinase; ERK, extracellular signal-regulated kinase; DC, dehydrocorydaline chloride)

To investigate the molecular mechanisms underlying the anti-proliferative effects of PnNVs on OSCC cells, we performed transcriptomic profiling PnNVs-treated LN4 cells. Heatmap visualization revealed widespread transcriptional alterations (Figure S4A). Gene ontology (GO) enrichment analysis indicated that the differentially expressed genes (DEGs) were primarily associated with chemokine-mediated signaling and the mitogen-activated protein kinase (MAPK) signaling pathway (Figure S4B). GSEA further confirmed significant enrichment of these two pathways (Fig. 4A, 4 F).

We first examined whether changes in the chemokine signaling pathway contribute functionally to PnNVs-induced cytotoxicity. RT-qPCR and western blot analysis confirmed the downregulation of several key chemokine genes and proteins, including C–C motif chemokine ligand 5 (CCL5) and C-X-C motif chemokine ligand 10 (CXCL10) (Fig. 4B–C). However, conditioned medium collected from PnNVs-treated LN4 cells failed to induce cytotoxicity in untreated cells, as shown by PI and PE staining assays (Fig. 4D–E). These results suggest that although PnNVs suppress chemokine-mediated intercellular signaling, this pathway is unlikely to be the primary driver of cell death. In contrast, the MAPK signaling pathway appeared functionally involved in PnNVs-mediated cytotoxicity. Transcriptomic and protein-level analyses revealed significant suppression of MAPK pathway components, including p-p38 and p-ERK (Fig. 4G–H). Importantly, treatment with dehydrocorydaline chloride (DC) [48], a MAPK agonist, partially rescued the cell death phenotype induced by PnNVs (Fig. 4I–J), indicating that MAPK inhibition contributes to the anti-proliferative effects of PnNVs.

**Fig. 5:**
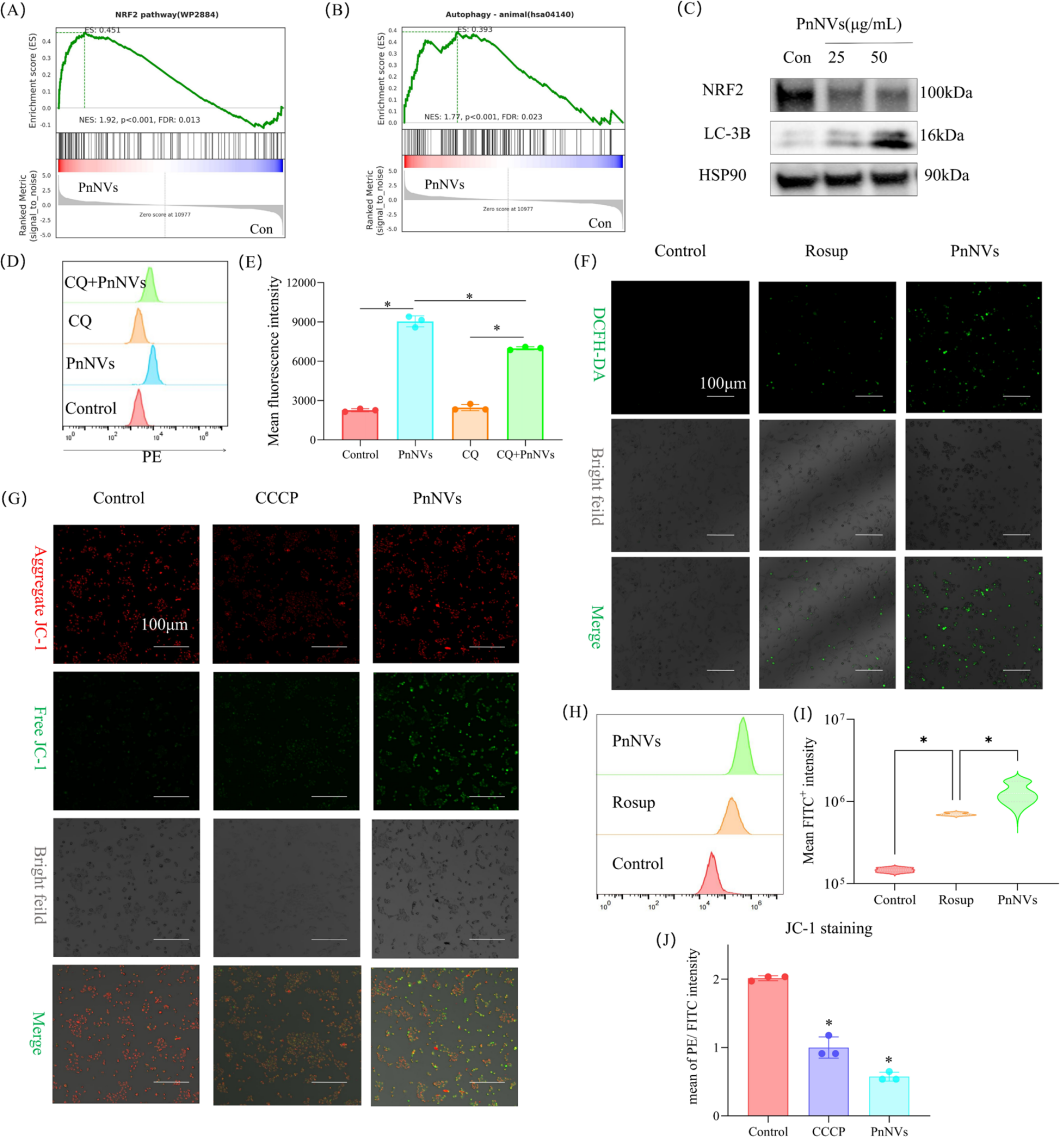
PnNVs induce ROS-dependent autophagic cell death through suppression of MAPK and NRF2 signaling. (**A–B**) GSEA plots showing enrichment of the NRF2 (**A**) and autophagy (**B**) pathways among DEGs in PnNVs-treated cells. (**C**) Western blot showing reduced NRF2 expression and increased LC3B levels after PnNVs treatment. (**D–E**) PI staining peak distribution (**D**) and PE fluorescence intensity (**E**) showing that autophagy inhibition with CQ attenuates PnNVs-induced cell death. (**F**) Fluorescence microscopy images of DCFH-DA–stained cells showing elevated ROS levels following PnNVs treatment. ROSup was included as a positive control. (**G**) JC-1 staining showing mitochondrial membrane depolarization in PnNVs-treated cells. (**H–I**) Flow cytometry analysis of ROS accumulation: (**H**) fluorescence peak shift and (**I**) quantification of mean fluorescence intensity. (**J**) Flow cytometry analysis of mitochondrial depolarization in JC-1–labeled cells. (Abbreviations: PnNVs, *Panax notoginseng*-derived nanovesicles; MAPK, mitogen-activated protein kinase; NRF2, nuclear factor erythroid 2–related factor 2; DEGs, differentially expressed genes; LC3B, microtubule-associated protein 1A/1B-light chain 3 beta; CQ, chloroquine; DCFH-DA, 2′,7′-dichlorodihydrofluorescein diacetate; ROS, reactive oxygen species; ROSup, positive control for ROS production; JC-1, 5,5′,6,6′-tetrachloro-1,1′,3,3′-tetraethylbenzimidazolylcarbocyanine iodide; PI, propidium iodide; PE, phycoerythrin channel)

Given that our previous results showed that PnNVs induce PANoptosis and ferroptosis in OSCC cells—both of which can be triggered by disrupted cellular redox homeostasis [49]—we next investigated the role of reactive oxygen species (ROS). GSEA revealed enrichment of nuclear factor erythroid 2–related factor 2 (NRF2) and autophagy-related pathways (Fig. 5A-B). Western blot analysis demonstrated a reduction in NRF2 expression and an increase in microtubule-associated protein 1A/1B-light chain 3 beta (LC3B) levels following PnNVs treatment, indicating activation of autophagy (Fig. 5C). Functional inhibition of autophagy using chloroquine (CQ) [50], a lysosomal inhibitor, partially reversed PnNVs-induced cell death (Fig. 5D-E), confirming the involvement of autophagy in this process.

Furthermore, 2',7'-dichlorodihydrofluorescein diacetate (DCFH-DA) staining followed by fluorescence microscopy and flow cytometry revealed significant ROS accumulation in PnNVs-treated cells (Fig. 5F, 5H–I). 5,5′,6,6′-Tetrachloro-1,1′,3,3′-tetraethylbenzimidazolylcarbocyanine iodide (JC-1) staining indicated a marked loss of mitochondrial membrane potential, implicating mitochondrial dysfunction as a major source of ROS (Fig. 5G, 5 J). Collectively, these results demonstrate that PnNVs induce ROS-dependent autophagic cell death in OSCC cells in association with reduced phosphorylation of p38 MAPK and decreased NRF2 expression. These findings highlight the MAPK/NRF2–ROS–autophagy axis as a potential mechanism contributing to the antitumor effects of PnNVs.

**Fig. 6:**
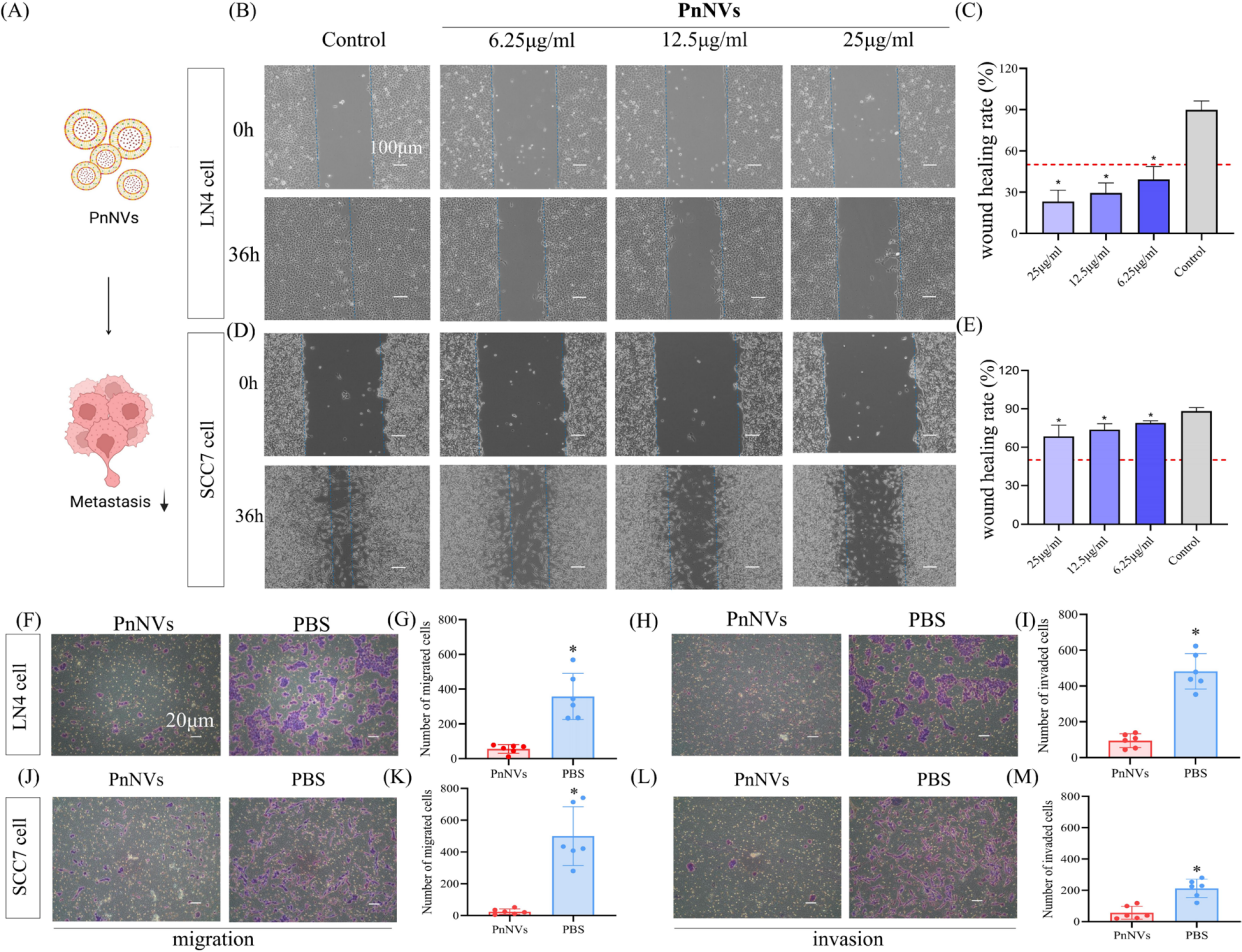
PnNVs regulate OSCC metastatic potential. (**A**) Schematic illustration showing the proposed mechanism by which PnNVs suppress OSCC metastasis. (**B–C**) Scratch wound healing assay showing reduced migratory capacity of LN4 cells after PnNVs treatment. (**D–E**) Scratch wound healing assay showing reduced migratory capacity of SCC7 cells after PnNVs treatment. (**F–G**) Transwell migration assay showing decreased migration of LN4 cells after PnNVs exposure. (**H–I**) Transwell invasion assay showing suppressed invasion following PnNVs intervention. (**J–K**) Transwell migration assay showing decreased migration of SCC7 cells after PnNVs exposure. (**L–M**) Transwell invasion assay showing suppressed invasion following PnNVs intervention. (Abbreviations: OSCC, oral squamous cell carcinoma; H&E, hematoxylin and eosin; PnNVs, *Panax notoginseng*-derived nanovesicles)

### PnNVs suppress OSCC cell dissemination by modulating adhesion and disrupting chemokine-mediated migration

To investigate the effect of PnNVs on the metastatic potential of OSCC cells, we first assessed their influence on cell motility (Fig. 6A). In scratch wound healing assays, PnNVs significantly inhibited the migratory capacity of LN4 and SCC7 cells even at low concentrations (Fig. 6B-E), indicating a robust and stable anti-migratory effect. These findings were further supported by transwell migration and invasion assays, where PnNVs-treated cells exhibited markedly reduced penetration through both uncoated and Matrigel-coated membranes (Fig. 6F-M).

**Fig. 7:**
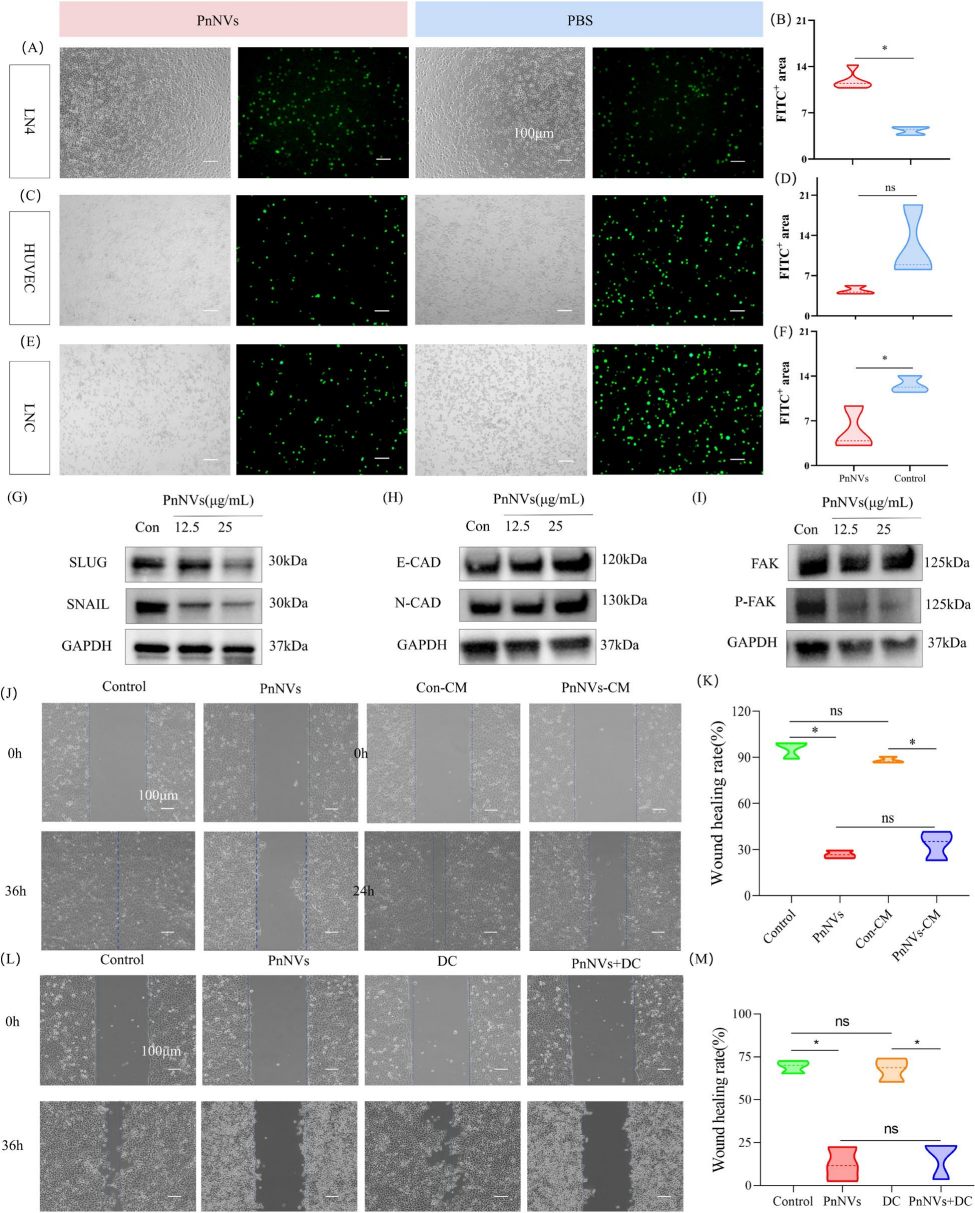
PnNVs inhibit OSCC cell metastasis by modulating adhesion and disrupting chemokine-mediated migration. (**A–B**) Cell–cell adhesion among LN4 cells after PBS or PnNVs treatment. (**C–D**) Adhesion between LN4 cells and HUVECs. (**E–F**) Adhesion between LN4 cells and LNCs. (**G–H**) Western blot analysis of EMT-related markers (SNAIL, SLUG, E-cadherin, N-cadherin) in LN4 cells after PnNVs treatment. (**I**) Western blot analysis of FAK expression as a marker of adhesion-related signaling. (**J–K**) Wound healing assay showing that CM from PnNVs-treated cells impaired the migratory capacity of untreated LN4 cells. (**L–M**) Wound healing assay showing that co-treatment with MAPK activator DC did not restore migration in PnNVs-treated LN4 cells. (Abbreviations: HUVECs, human umbilical vein endothelial cells; LNCs, lymphatic endothelial cells; EMT, epithelial–mesenchymal transition; FAK, focal adhesion kinase; CM, conditioned medium; MAPK, mitogen-activated protein kinase; DC, dehydrocorydaline chloride)

Given that alterations in signaling pathways alone may not fully account for the observed impairment in migration, we next turned our attention to cell adhesion behaviors, which play a pivotal role in metastasis by regulating tumor–tumor and tumor–endothelium interactions [51, 52]. PnNVs significantly enhanced homotypic adhesion among LN4 cells (Fig. 7A-B), while simultaneously reducing their adhesion to vascular endothelial cells (HUVECs) and lymphatic endothelial cells (LNCs) (Fig. 7C-F). This dual regulatory effect suggests that PnNVs may stabilize tumor cell clustering while also impeding dissemination into distant tissues.

To further elucidate the molecular basis of this adhesion remodeling, we analyzed key transcription factors associated with epithelial–mesenchymal transition (EMT) as well as core components of adhesion signaling. PnNVs treatment significantly downregulated the expression of EMT inducers SLUG [53] and SNAIL [54], while E-cadherin and N-cadherin levels remained largely unchanged (Fig. 7G-H). In addition, focal adhesion kinase (FAK) [55, 56] —a critical regulator of cell–substrate adhesion and cytoskeletal stability—was reduced following PnNVs exposure (Fig. 7I), suggesting that enhanced adhesion dynamics may contribute to the suppression of cell migration and dissemination.

Based on these observations, we further explored the underlying molecular mechanisms, focusing on chemokine and MAPK signaling pathways, which were among the most significantly downregulated in our transcriptomic analysis. Conditioned medium (CM) derived from PnNVs-treated cells significantly impaired the migration of untreated LN4 cells in scratch assays (Fig. 7J-K), suggesting that PnNVs may interfere with chemokine-mediated intercellular communication and thereby attenuate collective cell migration. Although MAPK signaling was concurrently suppressed (Fig. 4F-H), co-treatment with the MAPK agonist DC failed to restore the migratory ability of PnNVs-treated cells (Fig. 7L-M), indicating that MAPK inhibition does not play a central role in mediating the anti-migratory effects of PnNVs.

Collectively, these results demonstrate that PnNVs inhibit OSCC cell dissemination through a dual mechanism: by enhancing homotypic tumor cell adhesion to limit detachment and dispersion, and by disrupting chemokine-mediated communication and tumor–endothelial adhesion to impair the metastatic cascade. These findings reveal a multifaceted regulatory role for PnNVs in tumor cell migration and provide a theoretical basis for their potential as an anti-metastatic therapeutic strategy.

### Multi-omic profiling reveals synergistic RNA-metabolite cargos underlying the multi-targeted anticancer effects of PnNVs

**Fig. 8:**
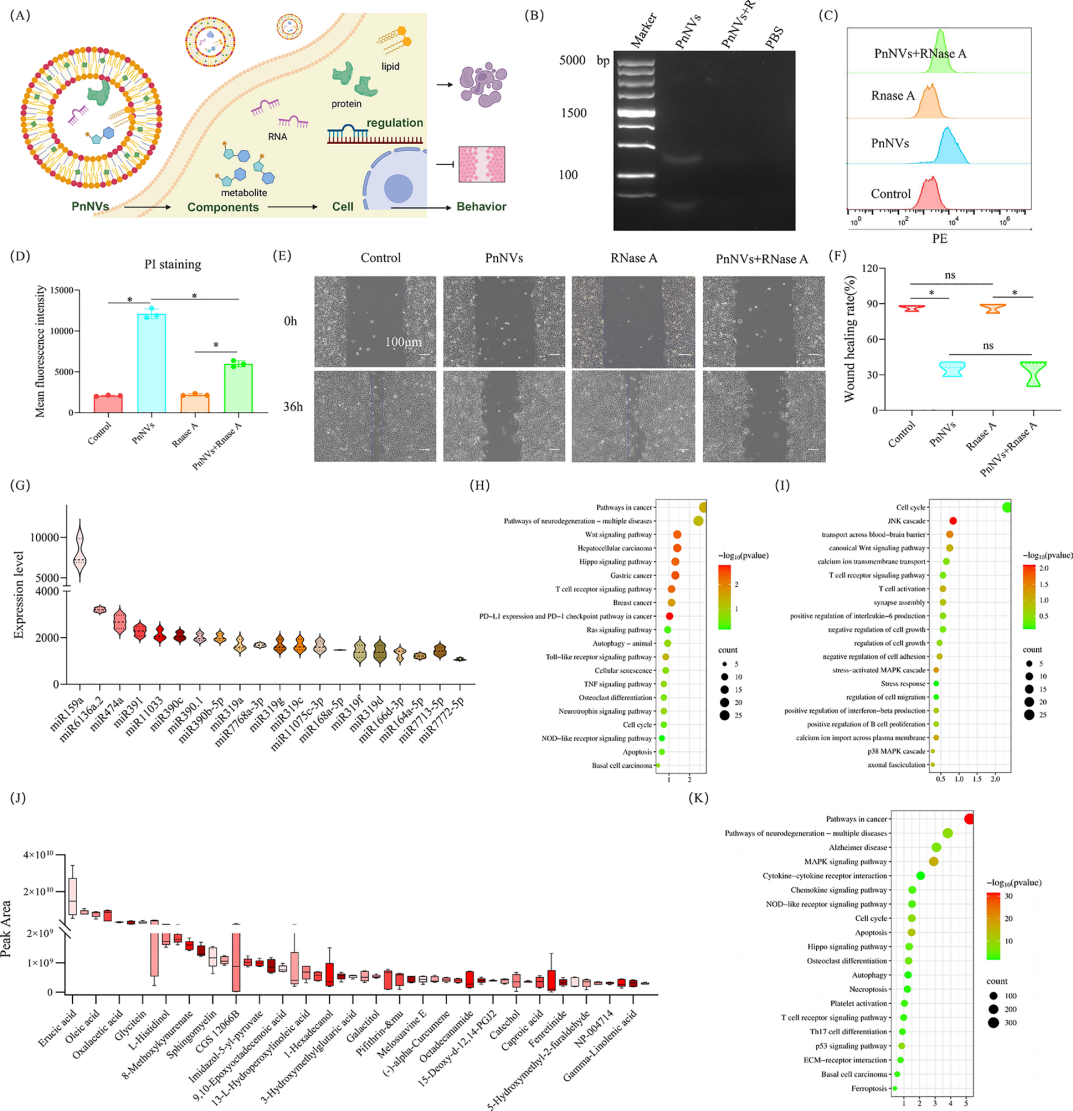
Multi-omic dissection of PnNVs cargos underlying OSCC suppression. (**A**) Schematic illustration of the multi-component regulatory effects of PnNVs on OSCC malignant phenotypes. (**B**) Agarose gel electrophoresis confirming RNA content in PnNVs before and after RNase A treatment. (**C**, **D**) Flow cytometry analysis and quantification of apoptotic LN4 cells treated with control or RNase-treated PnNVs. (**E**, **F**) Wound healing assays and quantification of migration in LN4 cells treated with control or RNase-treated PnNVs. (**G**) Top 20 most abundant miRNAs identified in PnNVs. (**H**) GO enrichment of predicted target genes of the top 20 miRNAs. (**I**) KEGG pathway enrichment of the top 20 miRNAs. (**J**) Top 50 metabolites identified by untargeted metabolomics. (**K**) GO enrichment of target genes associated with high-abundance metabolites

PnNVs exert multi-faceted antitumor activities against OSCC, yet the functional cargos responsible for these effects remain to be fully elucidated. PDNVs are known to contain diverse biomolecules, including metabolites, lipids, RNAs, and proteins [21]. Upon uptake, these cargos are released into the cytoplasm, where they regulate gene and protein expression, ultimately reshaping cellular behaviors (Fig. 8A). To dissect the active constituents, we systematically examined PnNVs after selective depletion of distinct cargo classes.

Proteinase K treatment effectively removed vesicular proteins (Figure S5A). Strikingly, protein depletion had no impact on the pro-apoptotic or anti-migratory properties of PnNVs, suggesting proteins are dispensable for these effects (Figure S4B, S5C, S5E, and S5F). Similarly, total lipid extracts (Figure S4H) failed to reproduce the cytotoxic or anti-migratory phenotypes of intact vesicles, indicating that lipids alone are insufficient drivers (Figure S5I-L).

We next focused on RNA cargos. Agarose gel electrophoresis confirmed abundant RNA in PnNVs, which was efficiently depleted by RNase A treatment (Fig. 8B). RNase-treated vesicles displayed a markedly reduced ability to trigger apoptosis in LN4 cells, highlighting a key role for RNA in the anti-proliferative activity of PnNVs (Fig. 8C and 8D). However, RNase treatment did not abolish their anti-migratory effects, implying that RNA is not the primary determinant of metastasis suppression (Fig. 8E-8F). Given the regulatory capacity of microRNAs (miRNAs) ^[57,58]^, we performed miRNA sequencing and identified a wide repertoire of vesicular miRNAs (Fig. 9G). GO enrichment of their predicted targets revealed associations with cell cycle regulation, proliferation, and differentiation (Fig. 8H). KEGG analysis further showed enrichment in signaling cascades such as p38 MAPK (Fig. 8I), aligning with the cellular phenotypes observed.

Considering these results, we hypothesized that metabolites may also contribute to PnNVs’ activity. Untargeted metabolomic profiling revealed a broad spectrum of metabolites (Fig. 8J). Functional enrichment of predicted metabolite–target interactions implicated pathways linked to cell cycle arrest, apoptosis, adhesion, necrosis, MAPK signaling, autophagy, and ferroptosis (Fig. 8K). These findings mirror the effects observed in functional assays.

Collectively, the data support a model in which PnNVs act through synergistic interactions between miRNAs and metabolic cargos. This combinatorial regulation fine-tunes oncogenic gene networks, thereby coordinating the suppression of OSCC proliferation and migration.

## Discussion

PDNVs have emerged as a promising class of natural bio-nanomaterials due to their wide availability, favorable biocompatibility, and intrinsic cargo of bioactive compounds [21, 59]. While prior studies have largely focused on their anti-inflammatory potential, modulation of the gut microbiome, or their utility as drug delivery vehicles [60, 61], the intrinsic anti-tumor properties of PDNVs remain underexplored—particularly in the context of aggressive solid tumors such as OSCC. To address this gap, we systematically screened PDNVs isolated from ten medicinal plants, guided by both traditional Chinese pharmacopeia and modern pharmacological literature. Among these, PnNVs exhibited the most potent anti-OSCC activity. Building on the observation that PnNVs significantly inhibited OSCC cell proliferation and migration both in vitro and in vivo (Fig. 1), we further integrated transcriptomic, miRNA, and metabolomic analyses with cargo depletion experiments to construct a mechanistic framework. This framework delineates a coordinated, multi-target anti-cancer mechanism exerted by PnNVs. By shifting the research paradigm from observational phenotypes to mechanistic dissection, our study not only advances the fundamental understanding of PDNVs but also establishes a novel translational strategy for applying plant-derived nanotherapeutics in solid tumor treatment.

The anti-tumor efficacy of PnNVs was validated through both in vitro functional assays and an orthotopic mouse model of tongue carcinoma. At the cellular level, PnNVs treatment significantly reduced OSCC cell proliferation (Fig. 3), impeded scratch closure, and suppressed migration (Fig. 6), indicating their potential for early-phase intervention against tumor cell motility. In vivo, PnNVs administration led to markedly reduced tumor burden and lymphatic dissemination in mice implanted with highly metastatic LN4-luc-GFP OSCC cells (Fig. 1F and 2C, 2D, and 2G). Notably, a subset of PnNVs-treated animals exhibited complete absence of lymph node metastasis. Furthermore, biodistribution analysis revealed a degree of PnNVs accumulation in tumor tissue (Fig. 2J–K), suggesting a passive tumor tropism. Although not equivalent to the enhanced permeability and retention (EPR) effect observed in chemotherapeutics, this enrichment underscores the dual potential of PnNVs as both innate therapeutics and engineerable carriers. Taken together, these results highlight PnNVs as a biocompatible, dual-functional platform capable of local enrichment and molecular intervention, offering a natural and low-toxicity therapeutic strategy for OSCC.

Programmed cell death represents a central therapeutic target in anti-cancer strategies [62, 63]; however, solid tumor cells often exhibit adaptive resistance to monotypic death pathways [64, 65]. In this study, we systematically demonstrated that PnNVs induce OSCC cell death through a multifaceted mechanism involving PANoptosis, ferroptosis, and a ROS–autophagy axis—forming a coordinated “multi-pathway lethal network” to overcome tumor resilience.

We first identified that PnNVs activate PANoptosis, a recently defined, integrated form of programmed cell death that encompasses features of apoptosis, necroptosis, and pyroptosis [66–68]. Western blotting revealed significant upregulation of cleaved caspase-3, phosphorylated-MLKL, cleaved-GSDMD, and cleaved GSDME upon PnNVs treatment, accompanied by increased expression of the upstream sensor ZBP1 (Fig. 3I), indicating the assembly of a PANoptosome complex [69]. This integrated death pathway is particularly effective against heterogeneous cell populations and can bypass apoptotic tolerance, thus offering notable advantages for targeting aggressive solid tumors.

Secondly, we observed a classic oxidative stress-mediated cytotoxic profile. DCFH-DA and JC-1 staining indicated markedly elevated ROS levels and mitochondrial depolarization following PnNVs exposure (Fig. 5F-J). Concurrently, NRF2, the master antioxidant regulator [70], was significantly downregulated at the protein level, while the autophagy marker LC3B [71] was upregulated. Partial rescue of cell viability by chloroquine further supports the activation of a ROS-driven, autophagy-dependent death pathway (Fig. 5C-E). This ROS–NRF2–autophagy axis imposes metabolic strain and bypasses classical apoptosis, ultimately pushing tumor cells toward stress collapse and energy depletion.

Importantly, transcriptomic analysis further implicated ferroptosis as a third axis of PnNVs-induced cytotoxicity. Enrichment of ferroptosis-related pathways was identified in RNA-seq data, with concurrent downregulation of GPX4 and SLC7A11, hallmark indicators of ferroptotic activity [72]. Given that ferroptosis triggers cell death through lipid peroxidation independently of mitochondrial signaling, its coordination with PANoptosis and oxidative autophagy enables a layered and redundant execution mechanism. Collectively, these findings reveal that PnNVs do not rely on a singular mode of action, but rather orchestrate a convergent network of PANoptosis, ferroptosis, and ROS–autophagy signaling to enhance tumor cell sensitivity to death stimuli. This multi-axial strategy expands the mechanistic repertoire of PDNVs-induced tumor suppression and highlights the translational promise of natural vesicle-based therapeutics in overcoming drug resistance through multi-targeted cell fate disruption.

Traditionally, tumor metastasis is predominantly driven by the epithelial–mesenchymal transition (EMT), a process characterized by downregulation of epithelial markers such as E-cadherin and upregulation of mesenchymal markers such as N-cadherin, thereby facilitating loss of polarity and enhanced cellular motility [73, 74]. However, our study revealed that the anti-migratory effect of PnNVs on OSCC cells is not mediated via classical EMT modulation, but rather through an alternative mechanism involving enhanced adhesion and structural anchoring. Western blot analysis showed no significant changes in E-cadherin or N-cadherin protein levels following PnNVs treatment, suggesting that PnNVs do not induce a canonical EMT reversal. Nevertheless, the expression of key EMT transcriptional repressors, SNAIL and SLUG, was markedly downregulated (Fig. 8G-H), while focal adhesion kinase (FAK), a mediator of cell–cell and cell–matrix adhesion [75], was reduced (Fig. 7I). These changes imply that PnNVs enhance cellular adhesion strength rather than reprogram epithelial identity. This interpretation was further supported by fluorescence imaging, which showed tighter intercellular boundaries and more cohesive cell arrangements after treatment, indicative of reduced motility. These results suggest that PnNVs suppress tumor cell migration by promoting mechanical anchoring and cytoskeletal stabilization, rather than through phenotypic switching.

In OSCC, metastatic dissemination is shaped not only by tumor-intrinsic motility but also by the dynamic interplay between tumor cells and the lymphatic–vascular interface [76]. Lymphovascular invasion represents a pivotal step that enables tumor cells to access lymphatic channels and is strongly associated with occult lymph node metastasis, serving as one of the most reliable pathological predictors in clinical practice [77]. Moreover, metastatic or tumor-reactive lymph nodes undergo substantial microenvironmental remodeling, including lymphangiogenesis, high endothelial venule (HEV) restructuring, and shifts in immune-cell composition, collectively creating a permissive niche that facilitates tumor colonization and further dissemination [78]. In this context, our findings that PnNVs reduce the adhesion of OSCC cells to lymphatic and vascular endothelial cells—while simultaneously strengthening tumor cell–tumor cell cohesion (Fig. 7A-F)—suggest that PnNVs-mediated anchoring may hinder the initial intravasation step required for lymphatic spread. These adhesion-related alterations, together with the suppression of key chemokine gradients, indicate that interventions capable of disrupting migratory cues or modifying cell–endothelium interactions may indirectly influence the likelihood of lymph node involvement in OSCC. Importantly, tumor metastasis is not solely determined by intrinsic cellular motility but also by the intercellular chemokine signaling networks that mediate tumor–stroma–immune interactions [79, 80]. Chemokine axes such as CCL5–CCR5 and CXCL10–CXCR3 have been implicated in migration, immune evasion, and directional lymphatic invasion [81, 82]. To investigate whether PnNVs interfere with these pathways, we analyzed transcriptomic and GSEA data, which revealed significant downregulation of chemokine signaling following PnNVs treatment, with CCL5 and CXCL10 showing the most prominent suppression. qPCR and Western blotting confirmed that both chemokines were significantly downregulated at the mRNA and protein levels (Figs. 4B-C).

Functionally, conditioned media derived from PnNVs-treated OSCC cells failed to promote scratch wound healing in OSCC cells (Fig. 7J-K), despite lacking direct cytotoxicity. This indicates that PnNVs alter the tumor cell secretome, disrupting paracrine signaling essential for collective migration. This disruption of the “chemokine–receptor–paracrine” axis further reinforces the anti-metastatic effect of PnNVs and reveals their potential role in modulating tumor microenvironment communication.

As naturally derived, multi-component nanovesicles, PDNVs are known to contain complex bioactive cargo—including miRNAs, metabolites, proteins, and lipids [21]. Historically, mechanistic studies have predominantly adhered to a “dominant component” hypothesis, attributing biological effects to a single class of molecules, such as specific miRNAs or active phytochemicals [24, 29]. However, our study is the first to demonstrate through systematic cargo depletion experiments that the anti-tumor efficacy of PnNVs in OSCC does not rely on any single component, but rather emerges from the synergistic actions of multiple molecular entities, particularly RNA and metabolite classes.

We employed a series of component elimination and selective extraction strategies [83] to dissect the contribution of each cargo type. Functionally, RNase-treated PnNVs, which retained their vesicular morphology and size, exhibited a significant reduction in apoptosis-inducing capacity (Fig. 8B–D), highlighting the critical role of RNA cargo in regulating programmed cell death. In contrast, depletion of proteins and extraction of lipids did not notably impair PnNVs function, suggesting that metabolites are likely key bioactive contributors. Importantly, none of the depletion strategies resulted in a complete loss of function, reinforcing the notion that PnNVs activity is governed by a modular, network-based mechanism rather than a linear, single-target paradigm. This “multi-factor, multi-target, multi-pathway” model aligns with the pharmacological characteristics of natural botanical products and supports the emerging concept of PDNVs as modular multi-effector units. These insights not only broaden our understanding of PDNVs-mediated mechanisms but also set new benchmarks for the rational optimization, engineering, and quality assessment of plant-derived nanotherapeutics. Future research should prioritize the identification and integration of complementary functional modules, rather than focusing solely on the purification or enrichment of individual bioactives.

To further elucidate the regulatory landscape of PnNVs in OSCC, we conducted integrated multi-omics analyses, including transcriptomic, miRNA, and metabolomic profiling, and established a multidimensional map of enriched pathways. This cross-omics approach not only enhances the reliability of mechanistic insights but also reveals the systemic and complex nature of PDNVs-mediated tumor regulation. Transcriptomic and GSEA results showed that PnNVs significantly suppressed multiple cancer-associated pathways, including MAPK, cytokine signaling, and chemokine axes, all of which are intimately linked to tumor cell survival, migration, immune evasion, and stress adaptation [84, 85]. Of note, although MAPK signaling appeared to exert partial cytoprotective effects in some death contexts, its downstream pro-migratory functions were not restored, suggesting that MAPK is involved in PnNVs-induced cell death but is not the primary mediator of anti-migratory activity.

The miRNA sequencing analysis revealed a repertoire of enriched plant miRNAs potentially targeting key oncogenic pathways, including MAPK, NF-κB, and autophagy-related genes. These findings are highly consistent with our observed activation of PANoptosis and the ROS–autophagy axis. Complementary metabolomic profiling identified several enriched phytochemicals within PnNVs, including notoginsenosides Rg1, Re, and Rd, as well as various isoflavones. These compounds have been previously reported to regulate oxidative stress, autophagy, and ferroptosis, suggesting that they may serve as the chemical basis for the death-inducing phenotypes we observed.

Collectively, the multi-omics datasets converge to support a unified regulatory framework, wherein PnNVs modulate three major functional modules in OSCC: cell fate programming, motility suppression, and intercellular signaling disruption. This systemic model highlights the intrinsic advantages of PDNVs as natural, multi-target therapeutic agents and offers a structural blueprint for the future development of PDNVs-centric omics databases and predictive targeting platforms.

Against the backdrop of increasing demand for precise and low-toxicity cancer therapies [86], PnNVs demonstrated favorable pharmacological efficacy and biocompatibility, highlighting their translational potential. *Panax notoginseng*, the botanical source of PnNVs, is a well-documented medicinal herb with a long history of clinical use and established safety profiles [87]. In this study, we employed standardized and scalable extraction protocols, yielding vesicles with consistent morphology, size, and zeta potential—essential prerequisites for large-scale production and quality control. In our animal models, PnNVs administration did not cause significant weight loss or histopathological abnormalities in major organs (Fig. 2). Blood biochemistry and H&E staining also revealed no overt toxicity, underscoring their favorable biosafety profile. Additionally, in vivo imaging suggested that PnNVs exhibit a natural tendency to accumulate in tumor tissues (Fig. 2), likely owing to the compatibility between their membrane components and the tumor microenvironment—providing a foundation for future targeting modifications.

It is worth emphasizing that PDNVs intrinsically combine therapeutic and delivery functionalities [88]. This dual property allows for two distinct translational paths: (i) as standalone therapeutic agents, harnessing endogenous miRNAs and metabolites to exert multi-target anti-tumor effects [89]; and (ii) as biocompatible delivery platforms [23], amenable to surface functionalization or exogenous cargo loading for combination therapies involving small molecules or siRNAs. Such plasticity will significantly broaden the application landscape of PDNVs across nanomedicine, localized oral drug delivery, and immunotherapy.

In conclusion, PnNVs represent a highly promising candidate in the PDNVs pipeline, offering multiple translational advantages—from botanical resource origin and scalable manufacturing to multifunctional therapeutic potential and delivery compatibility. Despite the systematic elucidation of the multi-pathway anti-tumor mechanisms of PnNVs in OSCC and the validation of their functional effects at cellular, molecular, and in vivo levels, several critical issues remain to be addressed.

First, the mechanistic understanding of PnNVs cargo remains at a correlative level. Although RNA and metabolite depletion assays have preliminarily demonstrated a synergistic multi-component mechanism, the precise molecular targets and downstream pathways of specific miRNAs and metabolites have yet to be delineated. Future studies should incorporate CRISPR interference systems, miRNA mimics/inhibitors, and metabolic pathway blockers to dissect direct causal relationships between key components and phenotypic outcomes. Such approaches would facilitate the transition of PnNVs research from enrichment and prediction toward mechanistic precision and target validation.

Second, the in vivo biodistribution, retention kinetics, and clearance pathways of PDNVs require more temporally and spatially resolved investigation. While we employed DiR fluorescence labeling for preliminary biodistribution analysis, this method does not fully capture clinically relevant delivery dynamics. Advanced strategies such as near-infrared live imaging, spatial transcriptomics, and nanoparticle tracking could be employed to construct a multiscale delivery atlas encompassing “tissue–cell–subcellular” resolution, thereby deepening our systems-level understanding of PDNVs fate in vivo.

Third, the impact of the tumor immune microenvironment on PDNVs efficacy warrants further exploration. Our current study focused primarily on tumor cell-intrinsic phenotypes and did not examine whether PDNVs modulate tumor-associated macrophages (TAMs), dendritic cells (DCs), or T lymphocytes. Notably, our mechanistic data indicate that PnNVs-induced PANoptosis includes a pyroptotic component, and GSDMD-dependent pyroptosis is well known to trigger the release of pro-inflammatory cytokines such as IL-1β and IL-18, thereby activating both innate and adaptive immune responses. Multiple studies have demonstrated that nanoplatform-induced GSDMD/GSDME-mediated immunogenic cell death can enhance antigen presentation, promote dendritic-cell maturation, and synergize with PD-1/PD-L1 blockade to achieve improved antitumor efficacy [43, 45]. In parallel, *Panax notoginseng* and its bioactive constituents have been widely reported to possess immunoregulatory properties, including the modulation of cytokines such as TNF-α, IL-1β, IL-6, and IFN-γ [90]. These observations together suggest that PDNVs may not only promote antitumor immunity through the induction of immunogenic cell death but may also reshape the OSCC immune landscape via their intrinsic plant-derived immunomodulatory features. Integrating cytokine profiling, immune-cell activation analyses, and approaches such as single-cell RNA sequencing and immunohistochemistry will help delineate a more complete picture of PDNVs–immune system interactions.

In summary, while this study offers several innovative insights into the intrinsic mechanisms, synergistic cargo interactions, anti-metastatic effects, and paracrine modulation capacities of PDNVs, their clinical translation still requires overcoming key technical and validation challenges. Future efforts should simultaneously advance in four major directions—mechanistic refinement, cargo standardization, delivery optimization, and indication expansion—to accelerate the bench-to-bedside transition of PDNVs in cancer therapy.

## Conclusion

In summary, this study unveils a novel mechanistic framework through which PnNVs exert multifaceted anti-tumor effects against OSCC. By orchestrating a synergistic inhibition of chemokine-driven intercellular signaling, redox defense systems, and survival-related pathways, PnNVs effectively suppress tumor proliferation, migration, and lymphatic dissemination. The discovery that PnNVs induce ROS-mediated PANoptosis and autophagic cell death, independent of canonical EMT modulation, establishes them as unique bioactive nanotherapeutics capable of bypassing classical resistance mechanisms. Furthermore, our multi-omics-based cargo-function analyses highlight the inherent complexity and cooperative functionality of vesicular RNAs, metabolites, and proteins in modulating tumor behavior. Together, these findings position PnNVs as a promising natural nanoplatform for precision cancer therapy, offering both standalone efficacy and combinatorial potential with current therapeutic modalities. Future work aimed at dissecting specific cargo-target interactions and immunomodulatory roles will further accelerate their translational development in oral oncology and beyond.

## Supplementary Information

Additional file 1.

## Data Availability

All data supporting the findings of this study are available from the corresponding author upon reasonable request.

## References

[1] Tan Y, Wang Z, Xu M, Li B, Huang Z, Qin S, et al. Oral squamous cell carcinomas: state of the field and emerging directions. Int J Oral Sci. 2023;15(1):44. 37736748 10.1038/s41368-023-00249-w PMC10517027

[2] Zhou Y, Wang L, Liu M, Jiang H, Wu Y. Oral squamous cell carcinoma: insights into cellular heterogeneity, drug resistance, and evolutionary trajectories. Cell Biol Toxicol. 2025;41(1):101. 40504271 10.1007/s10565-025-10048-0 PMC12162747

[3] Huang J, Chan SC, Ko S, Lok V, Zhang L, Lin X, et al. Disease burden, risk factors, and trends of lip, oral cavity, pharyngeal cancers: a global analysis. Cancer Med. 2023;12(17):18153–64. 37519070 10.1002/cam4.6391 PMC10524054

[4] Mohamad I, Glaun MDE, Prabhash K, Busheri A, Lai SY, Noronha V, et al. Current treatment strategies and risk stratification for oral carcinoma. Am Soc Clin Oncol Educ Book. 2023;43:e389810. 37200591 10.1200/EDBK_389810

[5] Cai H, Zhu Y, Wang C, Zhang Y, Hou J. Neck nodal recurrence and survival of clinical T1–2 N0 oral squamous cell carcinoma in comparison of elective neck dissection versus observation: a meta-analysis. Oral Surg Oral Med Oral Pathol Oral Radiol. 2020;129(4):296–310. 32107184 10.1016/j.oooo.2019.10.012

[6] Yu YF, Cao LM, Li ZZ, Zhong NN, Wang GR, Xiao Y, et al. Frequency of lymph node metastases at different neck levels in patients with oral squamous cell carcinoma: a systematic review and meta-analysis. Int J Surg. 2025;111(1):1285–300. 39037727 10.1097/JS9.0000000000001953 PMC11745673

[7] Petit C, Lacas B, Pignon JP, Le QT, Grégoire V, Grau C, et al. Chemotherapy and radiotherapy in locally advanced head and neck cancer: an individual patient data network meta-analysis. Lancet Oncol. 2021;22(5):727–36. 33862002 10.1016/S1470-2045(21)00076-0

[8] Borse RH, Ramakrishnan K, Gandhi J, Dhankhar P, Chirovsky D. Cost-effectiveness of pembrolizumab for the first-line treatment of recurrent or metastatic head and neck squamous cell carcinoma in the United States. J Med Econ. 2022;25(1):954–65. 35765888 10.1080/13696998.2022.2095826

[9] Yamauchi M, Minesaki A, Ishida T, Sato Y, Okamura S, Shuto H, et al. Induction chemotherapy with 5-fluorouracil, cisplatin, and cetuximab in advanced head and neck squamous cell carcinoma. In Vivo. 2023;37(3):1275–80. 37103108 10.21873/invivo.13205 PMC10188000

[10] Le Tourneau C, Ghiani M, Cau MC, Depenni R, Ronzino G, Bonomo P, et al. First-line cetuximab + platinum-based therapy for recurrent/metastatic head and neck squamous cell carcinoma: a real-world observational study-ENCORE. Cancer Rep. 2023;6(5):e1804. 10.1002/cnr2.1804 PMC10172179 37069784

[11] Mesía R, Rivera F, Kawecki A, Rottey S, Hitt R, Kienzer H, et al. Quality of life of patients receiving platinum-based chemotherapy plus cetuximab first line for recurrent and/or metastatic squamous cell carcinoma of the head and neck. Ann Oncol. 2010;21(10):1967–73. 20335368 10.1093/annonc/mdq077 PMC2946862

[12] Ma Y, Guo C, Wang X, Wei X, Ma J. Impact of chemotherapeutic agents on liver microenvironment: oxaliplatin create a pro-metastatic landscape. J Exp Clin Cancer Res. 2023;42(1):237. 37697332 10.1186/s13046-023-02804-z PMC10494354

[13] Zhu L, Chen L. Progress in research on paclitaxel and tumor immunotherapy. Cell Mol Biol Lett. 2019;24:40. 31223315 10.1186/s11658-019-0164-y PMC6567594

[14] Qiu X, Qu Y, Guo B, Zheng H, Meng F, Zhong Z. Micellar paclitaxel boosts ICD and chemo-immunotherapy of metastatic triple negative breast cancer. J Control Release. 2022;341:498–510. 34883139 10.1016/j.jconrel.2021.12.002

[15] Hu SY, Qian JX, Yang SY, Andriani L, Liao L, Deng L, et al. Destabilization of microrchidia family CW-type zinc finger 2 via the cyclin-dependent kinase 1-chaperone-mediated autophagy pathway promotes mitotic arrest and enhances cancer cellular sensitivity to microtubule-targeting agents. Clin Transl Med. 2023;13(3):e1210. 36967563 10.1002/ctm2.1210 PMC10040724

[16] Hsu PY, Chen JL, Kuo SL, Wang WL, Jan FW, Yang SH, et al. San-Zhong-Kui-Jian-Tang exerts antitumor effects associated with decreased cell proliferation and metastasis by targeting ERK and the epithelial-mesenchymal transition pathway in oral cavity squamous cell carcinoma. Integr Cancer Ther. 2022;21:15347354221134921. 36404765 10.1177/15347354221134921 PMC9679344

[17] Shi Z, Song T, Wan Y, Xie J, Yan Y, Shi K, et al. A systematic review and meta-analysis of traditional insect Chinese medicines combined chemotherapy for non-surgical hepatocellular carcinoma therapy. Sci Rep. 2017;7(1):4355. 28659623 10.1038/s41598-017-04351-y PMC5489479

[18] Narayanan S, de Mores AR, Cohen L, Anwar MM, Lazar F, Hicklen R, et al. Medicinal mushroom supplements in cancer: a systematic review of clinical studies. Curr Oncol Rep. 2023;25(6):569–87. 36995535 10.1007/s11912-023-01408-2

[19] Kim SD, Kim JH, Kim DH, Park JH, Gong Y, Sun C, et al. Comprehensive evaluation of traditional herbal medicine combined with adjuvant chemotherapy on post-surgical gastric cancer: a systematic review and meta-analysis. Integr Cancer Ther. 2024;23:15347354231226256. 38281123 10.1177/15347354231226256 PMC10823854

[20] Chen X, He L, Zhang C, Zheng G, Lin S, Zou Y, et al. Exploring new avenues of health protection: plant-derived nanovesicles reshape microbial communities. J Nanobiotechnology. 2024;22(1):269. 38764018 10.1186/s12951-024-02500-w PMC11103870

[21] Chen X, Xing X, Lin S, Huang L, He L, Zou Y, et al. Plant-derived nanovesicles: harnessing nature’s power for tissue protection and repair. J Nanobiotechnology. 2023;21(1):445. 38001440 10.1186/s12951-023-02193-7 PMC10668476

[22] Liu H, Luo GF, Shang Z. Plant-derived nanovesicles as an emerging platform for cancer therapy. Acta Pharm Sin B. 2024;14(1):133–54. 38239235 10.1016/j.apsb.2023.08.033 PMC10792991

[23] Chen X, Ji S, Yan Y, Lin S, He L, Huang X, et al. Engineered plant-derived nanovesicles facilitate tumor therapy: natural bioactivity plus drug controlled release platform. Int J Nanomedicine. 2023;18:4779–804. 37635909 10.2147/IJN.S413831 PMC10460188

[24] Yan G, Xiao Q, Zhao J, Chen H, Xu Y, Tan M, et al. *Brucea javanica* derived exosome-like nanovesicles deliver miRNAs for cancer therapy. J Control Release. 2024;367:425–40. 38295998 10.1016/j.jconrel.2024.01.060

[25] Ma X, Chen N, Zeng P, He Y, Zhang T, Lu Y, et al. Hypericum Perforatum-derived exosomes-like nanovesicles: a novel natural photosensitizer for effective tumor photodynamic therapy. Int J Nanomedicine. 2025;20:1529–41. 39925681 10.2147/IJN.S510339 PMC11806729

[26] Yang M, Luo Q, Chen X, Chen F. Bitter melon derived extracellular vesicles enhance the therapeutic effects and reduce the drug resistance of 5-fluorouracil on oral squamous cell carcinoma. J Nanobiotechnology. 2021;19(1):259. 34454534 10.1186/s12951-021-00995-1 PMC8400897

[27] Cao M, Yan H, Han X, Weng L, Wei Q, Sun X, et al. Ginseng-derived nanoparticles alter macrophage polarization to inhibit melanoma growth. J Immunother Cancer. 2019;7(1):326. 31775862 10.1186/s40425-019-0817-4 PMC6882204

[28] Chen T, Ma B, Lu S, Zeng L, Wang H, Shi W, et al. Cucumber-derived nanovesicles containing cucurbitacin B for non-small cell lung cancer therapy. Int J Nanomedicine. 2022;17:3583–99. 35974872 10.2147/IJN.S362244 PMC9376005

[29] Liu J, Xiang J, Jin C, Ye L, Wang L, Gao Y, et al. Medicinal plant-derived mtDNA via nanovesicles induces the cGAS-STING pathway to remold tumor-associated macrophages for tumor regression. J Nanobiotechnology. 2023;21(1):78. 36879291 10.1186/s12951-023-01835-0 PMC9990354

[30] Chen Q, Li Q, Liang Y, Zu M, Chen N, Canup BSB, et al. Natural exosome-like nanovesicles from edible tea flowers suppress metastatic breast cancer via ROS generation and microbiota modulation. Acta Pharm Sin B. 2022;12(2):907–23. 35256954 10.1016/j.apsb.2021.08.016 PMC8897038

[31] Gan RH, Wei H, Xie J, Zheng DP, Luo EL, Huang XY, et al. Notch1 regulates tongue cancer cells proliferation, apoptosis and invasion. Cell Cycle. 2018;17(2):216–24. 29117785 10.1080/15384101.2017.1395534 PMC5884382

[32] Huang F, Pang J, Xu L, Niu W, Zhang Y, Li S, et al. Hedyotis diffusa injection induces ferroptosis via the Bax/Bcl2/VDAC2/3 axis in lung adenocarcinoma. Phytomedicine. 2022;104:154319. 35853302 10.1016/j.phymed.2022.154319

[33] Luo J, Chen QX, Li P, Yu H, Yu L, Lu JL, et al. *Lobelia chinensis* Lour inhibits the progression of hepatocellular carcinoma via the regulation of the PTEN/AKT signaling pathway in vivo and in vitro. J Ethnopharmacol. 2024;318(Pt A):116886. 37429502 10.1016/j.jep.2023.116886

[34] Zhang L, Ren B, Zhang J, Liu L, Liu J, Jiang G, et al. Anti-tumor effect of Scutellaria barbata D. Don extracts on ovarian cancer and its phytochemicals characterisation. J Ethnopharmacol. 2017;206:184–92. 28571726 10.1016/j.jep.2017.05.032

[35] Liu H, Li X, Duan Y, Xie JB, Piao XL. Mechanism of gypenosides of *Gynostemma pentaphyllum* inducing apoptosis of renal cell carcinoma by PI3K/AKT/mTOR pathway. J Ethnopharmacol. 2021;271:113907. 33556477 10.1016/j.jep.2021.113907

[36] Xu J, Zhang Z, Hu H, Yang Y, Xiao C, Xi L, et al. Synergistic antitumor effects of Peiminine and Doxorubicin on breast cancer through enhancing DNA damage via ZEB1. Biomed Pharmacother. 2024;173:116353. 38432128 10.1016/j.biopha.2024.116353

[37] Xia L, Liu X, Mao W, Guo Y, Huang J, Hu Y, et al. Panax notoginseng saponins normalises tumour blood vessels by inhibiting EphA2 gene expression to modulate the tumour microenvironment of breast cancer. Phytomedicine. 2023;114:154787. 37060724 10.1016/j.phymed.2023.154787

[38] Lu MK, Chang CC, Chao CH, Hsu YC. Structural changes, and anti-inflammatory, anti-cancer potential of polysaccharides from multiple processing of Rehmannia glutinosa. Int J Biol Macromol. 2022;206:621–32. 35217089 10.1016/j.ijbiomac.2022.02.112

[39] Liu R, Zhang X, Cai Y, Xu S, Xu Q, Ling C, et al. Research progress on medicinal components and pharmacological activities of polygonatum sibiricum. J Ethnopharmacol. 2024;328:118024. 38484952 10.1016/j.jep.2024.118024

[40] Mi XJ, Park HR, Dhandapani S, Lee S, Kim YJ. Biologically synthesis of gold nanoparticles using Cirsium japonicum var. maackii extract and the study of anti-cancer properties on AGS gastric cancer cells. Int J Biol Sci. 2022;18(15):5809–26. 36263176 10.7150/ijbs.77734 PMC9576503

[41] Baohong L, Zhongyuan L, Ying T, Beibei Y, Wenting N, Yiming Y, et al. Latex derived from *Ficus carica* L. inhibited the growth of NSCLC by regulating the caspase/gasdermin/AKT signaling pathway. Food Funct. 2023;14(4):2239–48. 36762489 10.1039/d2fo02284b

[42] Huang B, Wang H, Liu S, Hao M, Luo D, Zhou Y, et al. Palmitoylation-dependent regulation of GPX4 suppresses ferroptosis. Nat Commun. 2025;16(1):867. 39833225 10.1038/s41467-025-56344-5 PMC11746948

[43] Zhou YR, Dang JJ, Yang QC, Sun ZJ. The regulation of pyroptosis by post-translational modifications: molecular mechanisms and therapeutic targets. EBioMedicine. 2024;109:105420. 39476537 10.1016/j.ebiom.2024.105420 PMC11564932

[44] Xiao Y, Zhang T, Ma X, Yang QC, Yang LL, Yang SC, et al. Microenvironment-responsive prodrug-induced pyroptosis boosts cancer immunotherapy. Adv Sci Weinh. 2021;8(24):e2101840. 34705343 10.1002/advs.202101840 PMC8693073

[45] Yang Q, Ma X, Xiao Y, Zhang T, Yang L, Yang S, et al. Engineering prodrug nanomicelles as pyroptosis inducer for codelivery of PI3K/mTOR and CDK inhibitors to enhance antitumor immunity. Acta Pharm Sin B. 2022;12(7):3139–55. 35865097 10.1016/j.apsb.2022.02.024 PMC9293721

[46] Zhan C, Huang M, Yang X, Hou J. MLKL: functions beyond serving as the executioner of necroptosis. Theranostics. 2021;11(10):4759–69. 33754026 10.7150/thno.54072 PMC7978304

[47] Yang W, Xu Y, Liu S, Gao L, Li S, Xie X, et al. Mebendazole induces ZBP-1 mediated PANoptosis of acute myeloid leukemia cells by targeting TUBA1A and exerts antileukemia effect. J Adv Res. 2025. 10.1016/j.jare.2025.02.013. 39952321 10.1016/j.jare.2025.02.013 PMC12684942

[48] Wang H, Guo S, Gao H, Ding J, Li H, Kong X, et al. Myostatin regulates energy homeostasis through autocrine- and paracrine-mediated microenvironment communication. J Clin Invest. 2024. 10.1172/JCI178303. 38889010 10.1172/JCI178303 PMC11324308

[49] Wang Y, Chai C, Lin W, Cao J, Li Z, Jin Y, et al. Oxidative stress-mediated PANoptosis and ferroptosis: exploration of multimodal cell death triggered by an AIE-active nano-photosensitizer via photodynamic therapy. Theranostics. 2025;15(14):6665–85. 40585993 10.7150/thno.111635 PMC12203667

[50] Ferreira PMP, Sousa RWR, Ferreira JRO, Militão GCG, Bezerra DP. Chloroquine and hydroxychloroquine in antitumor therapies based on autophagy-related mechanisms. Pharmacol Res. 2021;168:105582. 33775862 10.1016/j.phrs.2021.105582

[51] Smart JA, Oleksak JE, Hartsough EJ. Cell adhesion molecules in plasticity and metastasis. Mol Cancer Res. 2021;19(1):25–37. 33004622 10.1158/1541-7786.MCR-20-0595 PMC7785660

[52] Thapa N, Wen T, Cryns VL, Anderson RA. Regulation of cell adhesion and migration via microtubule cytoskeleton organization, cell polarity, and phosphoinositide signaling. Biomolecules. 2023. 10.3390/biom13101430. 37892112 10.3390/biom13101430 PMC10604632

[53] Kim H, Lee SB, Myung JK, Park JH, Park E, Kim DI, et al. SLUG is a key regulator of epithelial-mesenchymal transition in pleomorphic adenoma. Lab Invest. 2022;102(6):631–40. 35145202 10.1038/s41374-022-00739-1

[54] Wang Y, Shi J, Chai K, Ying X, Zhou BP. The role of Snail in EMT and tumorigenesis. Curr Cancer Drug Targets. 2013;13(9):963–72. 24168186 10.2174/15680096113136660102 PMC4004763

[55] Zhao X, Guan JL. Focal adhesion kinase and its signaling pathways in cell migration and angiogenesis. Adv Drug Deliv Rev. 2011;63(8):610–5. 21118706 10.1016/j.addr.2010.11.001 PMC3132829

[56] Dawson JC, Serrels A, Stupack DG, Schlaepfer DD, Frame MC. Targeting FAK in anticancer combination therapies. Nat Rev Cancer. 2021;21(5):313–24. 33731845 10.1038/s41568-021-00340-6 PMC8276817

[57] Kloosterman WP, Plasterk RH. The diverse functions of microRNAs in animal development and disease. Dev Cell. 2006;11(4):441–50. 17011485 10.1016/j.devcel.2006.09.009

[58] Shi J, Zhou T, Chen Q. Exploring the expanding universe of small RNAs. Nat Cell Biol. 2022;24(4):415–23. 35414016 10.1038/s41556-022-00880-5 PMC9035129

[59] Wang R, Zhang Y, Guo Y, Zeng W, Li J, Wu J, et al. Plant-derived nanovesicles: promising therapeutics and drug delivery nanoplatforms for brain disorders. Fundam Res. 2025;5(2):830–50. 40242551 10.1016/j.fmre.2023.09.007 PMC11997602

[60] Kang M, Kang M, Lee J, Yoo J, Lee S, Oh S. *Allium tuberosum*-derived nanovesicles with anti-inflammatory properties prevent DSS-induced colitis and modify the gut microbiome. Food Funct. 2024;15(14):7641–57. 38953279 10.1039/d4fo01366b

[61] Niu G, Jian T, Gai Y, Chen J. Microbiota and plant-derived vesicles that serve as therapeutic agents and delivery carriers to regulate metabolic syndrome. Adv Drug Deliv Rev. 2023;196:114774. 36906231 10.1016/j.addr.2023.114774

[62] Peng F, Liao M, Qin R, Zhu S, Peng C, Fu L, et al. Regulated cell death (RCD) in cancer: key pathways and targeted therapies. Signal Transduct Target Ther. 2022;7(1):286. 35963853 10.1038/s41392-022-01110-y PMC9376115

[63] Qian S, Long Y, Tan G, Li X, Xiang B, Tao Y, et al. Programmed cell death: molecular mechanisms, biological functions, diseases, and therapeutic targets. MedComm. 2024;5(12):e70024. 39619229 10.1002/mco2.70024 PMC11604731

[64] Aldea M, Andre F, Marabelle A, Dogan S, Barlesi F, Soria JC. Overcoming resistance to tumor-targeted and immune-targeted therapies. Cancer Discov. 2021;11(4):874–99. 33811122 10.1158/2159-8290.CD-20-1638

[65] Crucitta S, Cucchiara F, Mathijssen R, Mateo J, Jager A, Joosse A, et al. Treatment-driven tumour heterogeneity and drug resistance: lessons from solid tumours. Cancer Treat Rev. 2022;104:102340. 35151155 10.1016/j.ctrv.2022.102340

[66] Yang QC, Wang YY, Wang S, Song A, Wang WD, Zhang L, et al. Engineered bacterial membrane biomimetic covalent organic framework as nano-immunopotentiator for cancer immunotherapy. Bioact Mater. 2025;47:283–94. 39925708 10.1016/j.bioactmat.2025.01.018 PMC11803166

[67] Hou K, Pan W, Liu L, Yu Q, Ou J, Li Y, et al. Molecular mechanism of PANoptosis and programmed cell death in neurological diseases. Neurobiol Dis. 2025;209:106907. 40204169 10.1016/j.nbd.2025.106907

[68] Bertheloot D, Latz E, Franklin BS. Necroptosis, pyroptosis and apoptosis: an intricate game of cell death. Cell Mol Immunol. 2021;18(5):1106–21. 33785842 10.1038/s41423-020-00630-3 PMC8008022

[69] Lee S, Karki R, Wang Y, Nguyen LN, Kalathur RC, Kanneganti TD. AIM2 forms a complex with pyrin and ZBP1 to drive PANoptosis and host defence. Nature. 2021;597(7876):415–9. 34471287 10.1038/s41586-021-03875-8 PMC8603942

[70] Rojo de la Vega M, Chapman E, Zhang DD. NRF2 and the hallmarks of cancer. Cancer Cell. 2018;34(1):21–43. 29731393 10.1016/j.ccell.2018.03.022 PMC6039250

[71] Kim S, Chun H, Kim Y, Kim Y, Park U, Chu J, et al. Astrocytic autophagy plasticity modulates Aβ clearance and cognitive function in Alzheimer’s disease. Mol Neurodegener. 2024;19(1):55. 39044253 10.1186/s13024-024-00740-w PMC11267931

[72] Zhang H, Pan J, Huang S, Chen X, Chang ACY, Wang C, et al. Hydrogen sulfide protects cardiomyocytes from doxorubicin-induced ferroptosis through the SLC7A11/GSH/GPx4 pathway by Keap1 S-sulfhydration and Nrf2 activation. Redox Biol. 2024;70:103066. 38359744 10.1016/j.redox.2024.103066 PMC10877437

[73] Mittal V. Epithelial mesenchymal transition in tumor metastasis. Annu Rev Pathol. 2018;13:395–412. 29414248 10.1146/annurev-pathol-020117-043854

[74] Allgayer H, Mahapatra S, Mishra B, Swain B, Saha S, Khanra S, et al. Epithelial-to-mesenchymal transition (EMT) and cancer metastasis: the status quo of methods and experimental models 2025. Mol Cancer. 2025;24(1):167. 40483504 10.1186/s12943-025-02338-2 PMC12144846

[75] Mitra SK, Hanson DA, Schlaepfer DD. Focal adhesion kinase: in command and control of cell motility. Nat Rev Mol Cell Biol. 2005;6(1):56–68. 15688067 10.1038/nrm1549

[76] Li ZZ, Zhou K, Wu Q, Liu B, Bu LL. Lymph node metastasis in cancer: clearing the clouds to see the dawn. Crit Rev Oncol Hematol. 2024;204:104536. 39426554 10.1016/j.critrevonc.2024.104536

[77] Huang S, Zhu Y, Cai H, Zhang Y, Hou J. Impact of lymphovascular invasion in oral squamous cell carcinoma: a meta-analysis. Oral Surg Oral Med Oral Pathol Oral Radiol. 2021;131(3):319-328.e1. 33309267 10.1016/j.oooo.2020.10.026

[78] Wei LY, Li ZZ, Xu ZY, Wang GR, Xiao Y, Liu B, et al. The ending is not the end: lymph node metastasis in oral squamous cell carcinoma. Int Immunopharmacol. 2025;146:113917. 39721451 10.1016/j.intimp.2024.113917

[79] Fares J, Fares MY, Khachfe HH, Salhab HA, Fares Y. Molecular principles of metastasis: a hallmark of cancer revisited. Signal Transduction and Targeted Therapy. 2020;5(1):28. 32296047 10.1038/s41392-020-0134-x PMC7067809

[80] Valastyan S, Weinberg RA. Tumor metastasis: molecular insights and evolving paradigms. Cell. 2011;147(2):275–92. 22000009 10.1016/j.cell.2011.09.024 PMC3261217

[81] Tokunaga R, Zhang W, Naseem M, Puccini A, Berger MD, Soni S, et al. CXCL9, CXCL10, CXCL11/CXCR3 axis for immune activation - a target for novel cancer therapy. Cancer Treat Rev. 2018;63:40–7. 29207310 10.1016/j.ctrv.2017.11.007 PMC5801162

[82] Guldner IH, Wang Q, Yang L, Golomb SM, Zhao Z, Lopez JA, et al. CNS-native myeloid cells drive immune suppression in the brain metastatic niche through Cxcl10. Cell. 2020;183(5):1234-1248.e25. 33113353 10.1016/j.cell.2020.09.064 PMC7704908

[83] Zhao Q, Feng J, Liu F, Liang Q, Xie M, Dong J, et al. *Rhizoma drynariae*-derived nanovesicles reverse osteoporosis by potentiating osteogenic differentiation of human bone marrow mesenchymal stem cells via targeting ERα signaling. Acta Pharm Sin B. 2024;14(5):2210–27. 38799625 10.1016/j.apsb.2024.02.005 PMC11119514

[84] Yi M, Li T, Niu M, Zhang H, Wu Y, Wu K, et al. Targeting cytokine and chemokine signaling pathways for cancer therapy. Signal Transduct Target Ther. 2024;9(1):176. 39034318 10.1038/s41392-024-01868-3 PMC11275440

[85] Bahar ME, Kim HJ, Kim DR. Targeting the RAS/RAF/MAPK pathway for cancer therapy: from mechanism to clinical studies. Signal Transduct Target Ther. 2023;8(1):455. 38105263 10.1038/s41392-023-01705-z PMC10725898

[86] Wang X, Li C, Wang Y, Chen H, Zhang X, Luo C, et al. Smart drug delivery systems for precise cancer therapy. Acta Pharm Sin B. 2022;12(11):4098–121. 36386470 10.1016/j.apsb.2022.08.013 PMC9643298

[87] Zhang S, Chen C, Lu W, Wei L. Phytochemistry, pharmacology, and clinical use of *Panax notoginseng* flowers buds. Phytother Res. 2018;32(11):2155–63. 30088301 10.1002/ptr.6167

[88] Li A, Li D, Gu Y, Liu R, Tang X, Zhao Y, et al. Plant-derived nanovesicles: further exploration of biomedical function and application potential. Acta Pharm Sin B. 2023;13(8):3300–20. 37655320 10.1016/j.apsb.2022.12.022 PMC10465964

[89] Corvigno S, Liu Y, Bayraktar E, Stur E, Bayram NN, Ahumada AL, et al. Enhanced plant-derived vesicles for nucleotide delivery for cancer therapy. NPJ Precis Oncol. 2024;8(1):86. 38582949 10.1038/s41698-024-00556-3 PMC10998889

[90] Luo H, Vong CT, Tan D, Zhang J, Yu H, Yang L, et al. *Panax notoginseng* saponins modulate the inflammatory response and improve IBD-like symptoms via TLR/NF-[Formula: see text]B and MAPK signaling pathways. Am J Chin Med. 2021;49(4):925–39. 33829964 10.1142/S0192415X21500440

