# Supplementary material for: Natural panax notoginseng-derived nanovesicles trigger multiple cell death mechanisms and reprogram chemokine signaling to impede oral squamous cell carcinoma progression
Source: J Nanobiotechnology. 2026 Mar 6;24:348. doi: 10.1186/s12951-026-04193-9 (PMC13081606; doi:10.1186/s12951-026-04193-9)
Supplement: Supplementary file 1 — Additional file 1. [file 12951_2026_4193_MOESM1_ESM.docx]

**Natural *Panax notoginseng*-derived Nanovesicles Trigger Multiple Cell Death Mechanisms and Reprogram Chemokine Signaling to Impede Oral Squamous Cell Carcinoma Proliferation and Metastasis**

Xiaohang Chen, Meifang Lin, Xuzhen Zhan, Genggeng Zheng, Shuoqi Lin, Liyu Huang, Chaochao Zhang, Yuxiang Yan, Hengyi Li, Zhaoyu Zhang, Xing Wang, Dali Zheng, and Youguang Lu


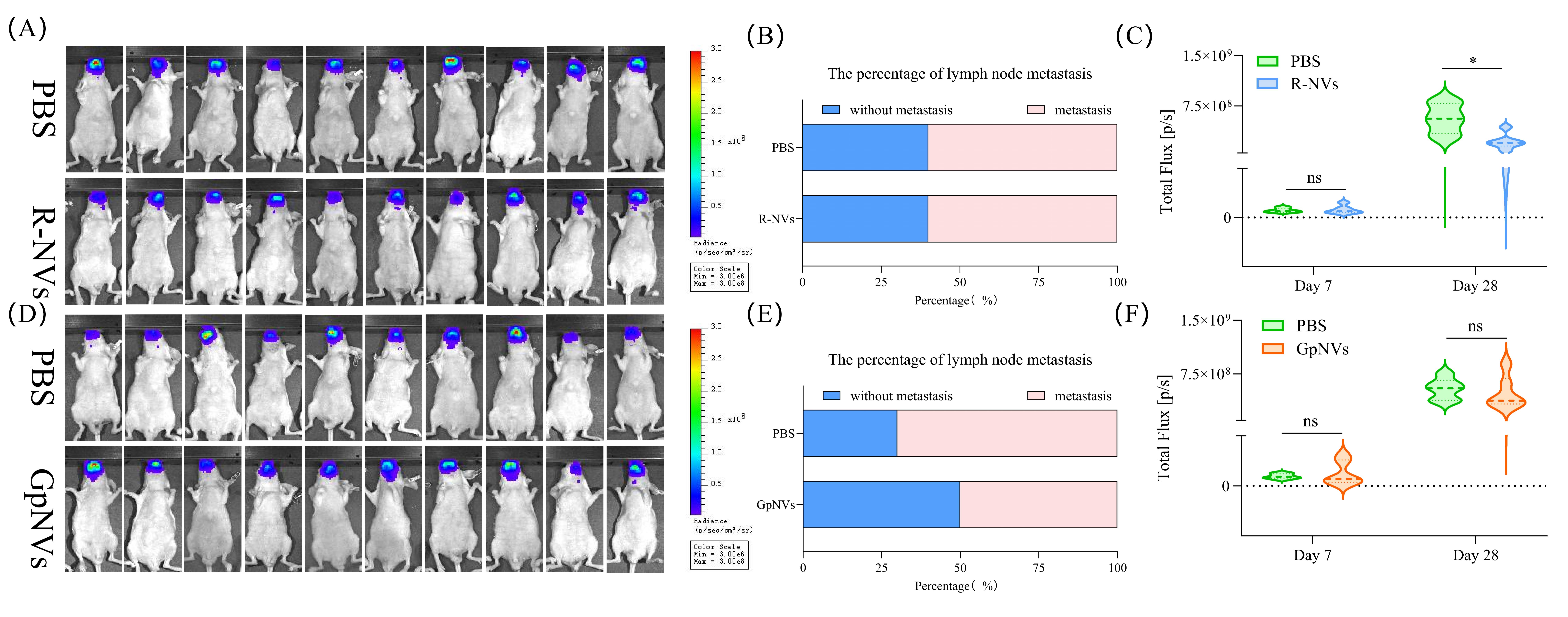


Figure S1. **Screening of PDNVs for anti-OSCC activity in vivo.** (A-C) Bioluminescence imaging (A), cervical lymph node metastasis incidence (B), and primary tumor fluorescence intensity (C) in OSCC-bearing mice treated with R-NVs. (D–F) Bioluminescence imaging (D), cervical lymph node metastasis incidence (E), and primary tumor fluorescence intensity (F) following treatment with GpNVs.( R-NVs, *Rehmannia glutinosa*-derived nanovesicles; GpNVs, *Gynostemma pentaphyllum*-derived nanovesicles.)


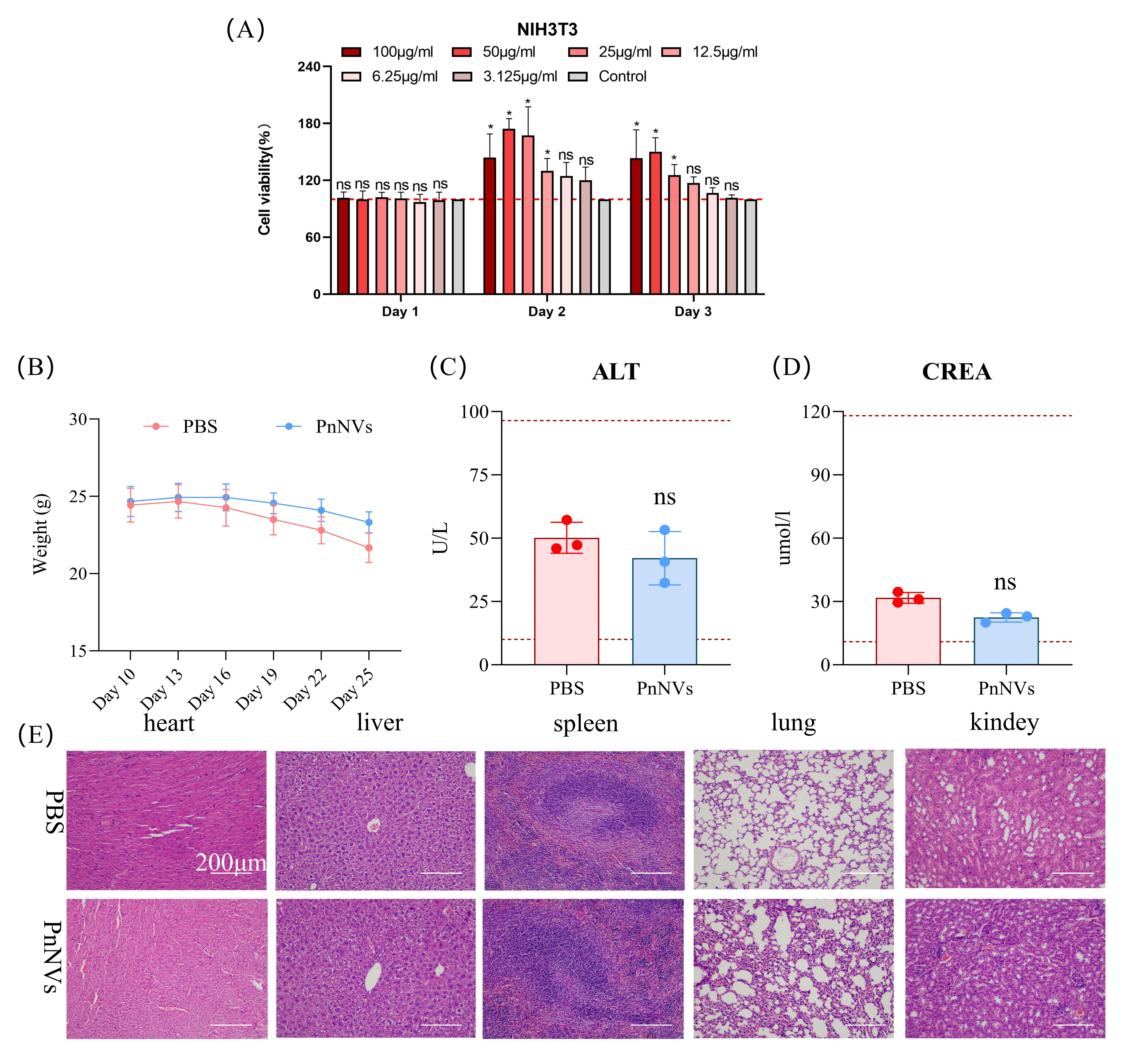


**Figure S2. Biocompatibility assessment of PnNVs.** (A–B) Viability of NIH3T3 fibroblasts (A) and human oral keratinocytes (B) treated with PnNVs at indicated concentrations for 1–3 days. (C) Body weight monitoring of tumor-bearing mice after repeated PnNV administration. (D–E) Serum alanine aminotransferase (ALT) (D) and creatinine (E) levels in mice treated with or without PnNVs. (F) Representative H&E staining of major organs (heart, liver, spleen, lungs, kidneys) from mice with or without PnNV treatment, showing no evident pathological changes.





**Figure S3. Effects of PnNVs on proliferation and cell death in OSCC cells.** (A) Representative EdU staining images showing reduced DNA synthesis in LN4 cells after PnNVs treatment. (B) Live/dead cell staining of LN4 cells following PnNVs treatment. (C) CCK-8 assay showing dose-dependent effects of PnNVs on SCC7 cell viability after 24 h and 48 h of treatment. (D) Representative EdU incorporation images of SCC7 cells after PnNV treatment (green: EdU; blue: nuclei). (E) Live/dead staining of SCC7 cells following PnNV exposure (green: live cells; red: dead cells). (F-G) Flow cytometry analysis showing PI fluorescence peak shifts in the PE channel (F) and quantification of PI intensity (G) after PnNV treatment. (H) GSEA enrichment plots showing activation of the ferroptosis pathway. (I) GSEA enrichment plots showing activation of the apoptosis pathway. (J) GSEA enrichment plots showing activation of the NOD-like receptor signaling pathway. K) GSEA enrichment plots showing activation of the necroptosis pathway. (Abbreviations: PnNVs, Panax notoginseng-derived nanovesicles; CCK-8, Cell Counting Kit-8; EdU, 5-ethynyl-2′-deoxyuridine; PI, propidium iodide; PE, phycoerythrin channel.)


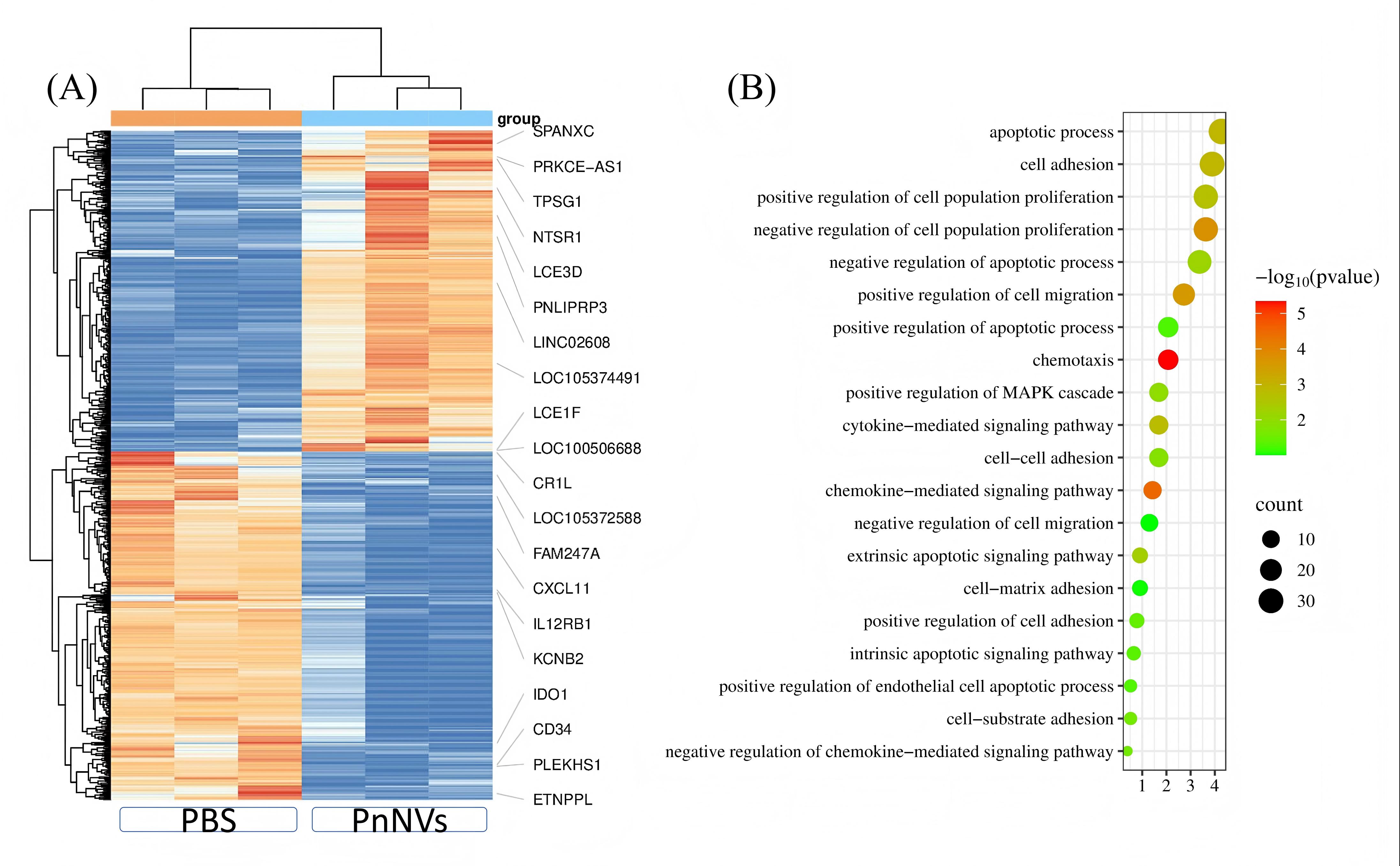


**Figure S4. Transcriptomic response of OSCC cells to PnNVs treatment.** (A) Heatmap showing differentially expressed mRNAs in LN4 cells after PnNV treatment. (B) GO enrichment analysis of DEGs, highlighting significantly affected biological processes. (Abbreviations: OSCC, oral squamous cell carcinoma; PnNVs, Panax notoginseng-derived nanovesicles; GO, Gene Ontology; DEGs, differentially expressed genes.)


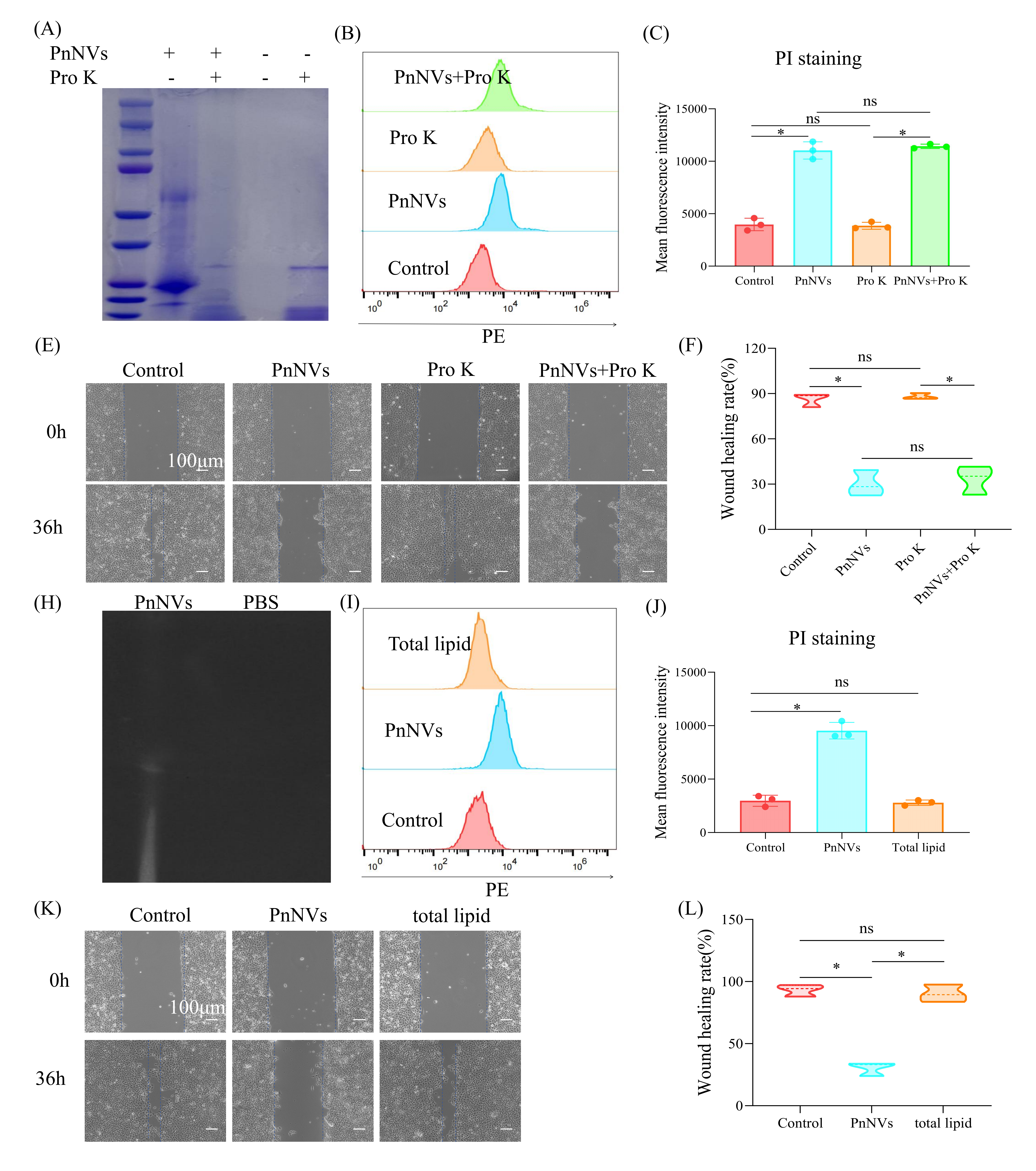


**Figure S5. Evaluation of the functional roles of proteins and lipids in PnNVs.** (A) SDS-PAGE analysis showing protein content in PnNVs before and after proteinase K treatment. (B) PI-based flow cytometry and (C) PE signal quantification in LN4 cells treated with protein-depleted vs. intact PnNVs. (E) Representative wound healing images and (F) quantification of cell migration following the same treatments.(H) Characterization of lipid components extracted from PnNVs. (I) PI flow cytometry, (J) PE signal quantification, (K) wound healing images, and (L) migration analysis following treatment with lipid extracts.
